# Comparative genomics of *Salmonella enterica* serovar Montevideo reveals lineage-specific gene differences that may influence ecological niche association

**DOI:** 10.1099/mgen.0.000202

**Published:** 2018-07-27

**Authors:** Scott V. Nguyen, Dayna M. Harhay, James L. Bono, Timothy P. L. Smith, Patricia I. Fields, Blake A. Dinsmore, Monica Santovenia, Rong Wang, Joseph M. Bosilevac, Gregory P. Harhay

**Affiliations:** ^1^​ UCD-Centre for Food Safety, School of Public Health, Physiotherapy and Sports Science, University College Dublin, Belfield, Dublin D04 N2E5, Ireland; ^2^​ USDA-ARS-US Meat Animal Research Center, Clay Center, NE 68933, USA; ^3^​ Enteric Disease Laboratory Branch, Division of Foodborne, Waterborne, and Environmental Diseases, Centers for Disease Control and Prevention, Atlanta, GA 30329, USA

**Keywords:** *Salmonella enterica* serovar Montevideo, comparative genomics, phylogenetic, CRISPR-Cas, cattle

## Abstract

*Salmonella enterica* serovar Montevideo has been linked to recent foodborne illness outbreaks resulting from contamination of products such as fruits, vegetables, seeds and spices. Studies have shown that Montevideo also is frequently associated with healthy cattle and can be isolated from ground beef, yet human salmonellosis outbreaks of Montevideo associated with ground beef contamination are rare. This disparity fuelled our interest in characterizing the genomic differences between Montevideo strains isolated from healthy cattle and beef products, and those isolated from human patients and outbreak sources. To that end, we sequenced 13 Montevideo strains to completion, producing high-quality genome assemblies of isolates from human patients (*n*=8) or from healthy cattle at slaughter (*n*=5). Comparative analysis of sequence data from this study and publicly available sequences (*n*=72) shows that Montevideo falls into four previously established clades, differentially occupied by cattle and human strains. The results of these analyses reveal differences in metabolic islands, environmental adhesion determinants and virulence factors within each clade, and suggest explanations for the infrequent association between bovine isolates and human illnesses.

## Data Summary

**Fig. 1.**
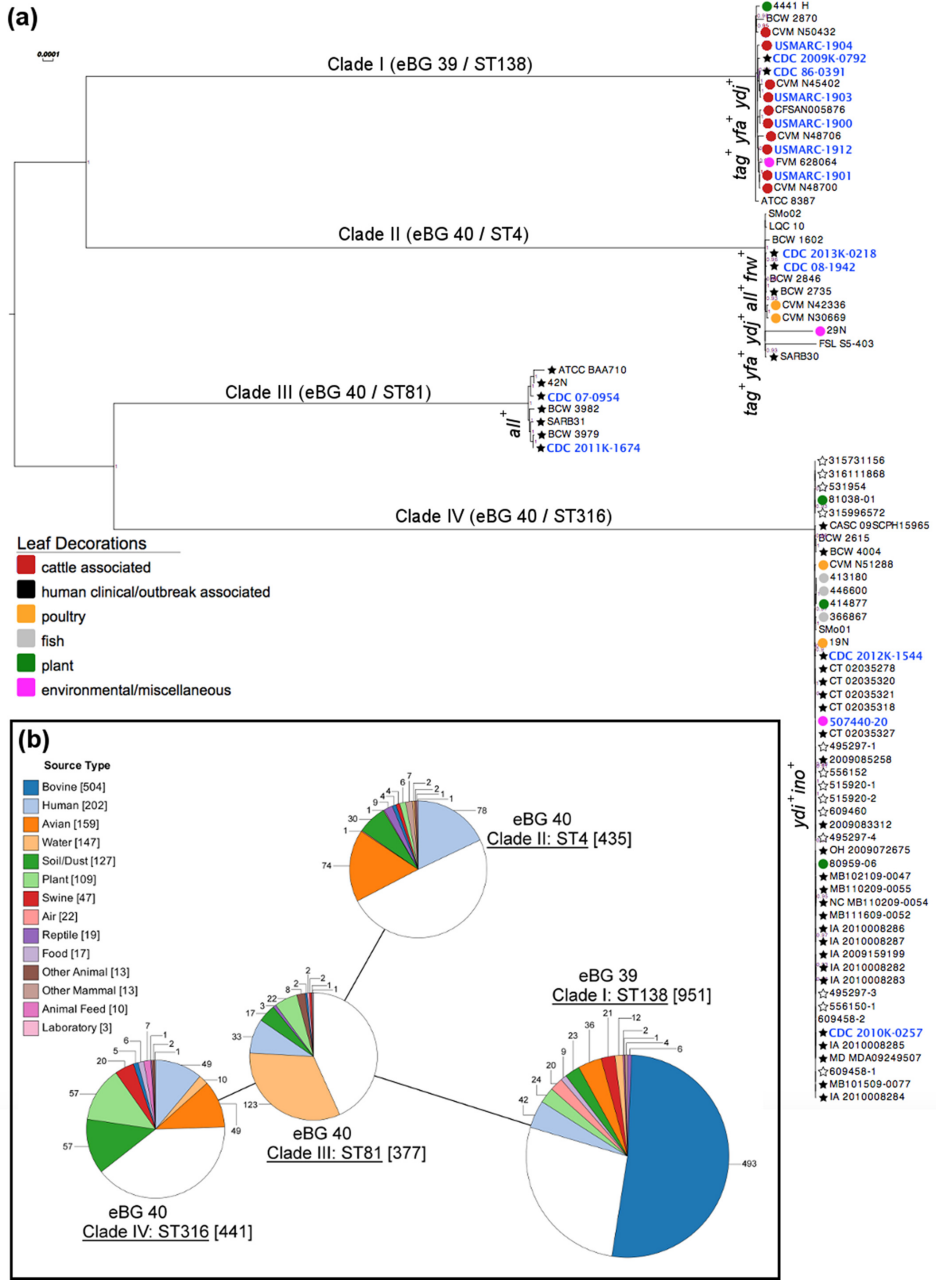
Phylogenetic and multilocus sequence typing (MLST) analysis of Montevideo strains. (a) Whole genome-based phylogenetic tree generated using Parsnp [25]. Strain names in blue font indicate complete and closed genomes including the 13 sequenced in this study. Filled stars represent strains isolated from human clinical cases, empty stars are strains associated with known foodborne outbreaks, and circles are associated with non-human sources. In addition, circles are coloured according to isolation source if known. Strains without symbols have unknown source metadata. Metabolic islands: *all –* allantoin, *frw –* fructose phosphotransferase, *tag –* tagatose phosphotransferase, *ino –* inositol, *ydi –* putative shikimate metabolic island, *ydj –* uncharacterized aldo/keto kinase major faciliator transport system, *yfa –* l-rhamnonate islet. (b) MLST analysis of 2204 Montevideo strains, and minimal spanning tree generated using MSTree v2 and visualized using GrapeTree, in EnteroBase [26]. Sequence type (ST) and eBG corresponding to each clade is indicated, along with the number of strains in each eBG attributed to a particular source type. Source type and number of strains attributed to each source are coloured according to the key. White pie slices indicate strains within a given ST where source type metadata were missing.

The GenBank accession numbers for all genomic sequence data analysed in this study and used to generate Fig. 1(a) can be found in Table S1 (available in the online version of this article). The PacBio base modification summary files used to examine DNA base modification were deposited in GenBank and the motifs and DNA methylases identified are available in REBASE (http://rebase.neb.com/rebase/rebase.html). CRISPR spacers were analysed and visualized using the CRISPR DB II Excel Macro developed by and available from Dr Philippe Horvath of DuPont. Geneious 10.1.3 was used to construct and query a *Salmonella* blast (blast v2.6.0) database derived from the Virulence Factor Database [1] to identify secreted effectors and fimbrial genes within each strain sequenced in this study. All phage regions identified by PHASTER analysis of the strains sequenced are listed in Table S2. The locus tag IDs for all putative adhesins, secreted effectors and other virulence factors identified by blast analysis may be found in Tables S3–S6.

**Impact Statement**

*Salmonella enterica* serovar Montevideo is consistently among the Center for Disease Control's list of top 20 serovars attributed to human salmonellosis in the USA. Surveys of *Salmonella* associated with cattle and beef have shown Montevideo to be a common serotype of this commodity, yet salmonellosis outbreaks attributed to ground beef contamination with Montevideo are extremely rare. We used single molecule real time sequencing to construct complete genome sequences of Montevideo isolated from healthy cattle, beef products and human patients in order to examine genomic differences between them. Phylogenetic analysis of these, and publicly available sequence data, showed strains of this serotype fall into four distinct clades. While human isolates were found in all four clades, bovine isolates were restricted to one clade. Our analysis revealed distinct differences in gene content among members of the four clades, especially with regard to prophage distribution, secreted effector and virulence factors, and fimbrial operon content. Moreover, our results suggest that different combinations of the adaptive (CRISPR) and innate (R-M) immune systems and the resulting differences in genome plasticity have played a marked role in the diversification of members of this serovar.

## Introduction

*Salmonella enterica* subsp*. enterica* are an important group of human, agricultural and foodborne pathogens. Non-typhoidal *Salmonella* (NTS) infections are estimated to account for over 1 million cases annually and result in over 19 000 hospitalizations [2]. While over 2600 serovars of *Salmonella* have been identified, only a limited number of serovars are responsible for the majority of NTS human illnesses [3]. With the advent of next-generation sequencing, there has been a deluge of genomic sequencing data, especially for *Salmonella enterica,* with 7842 publicly available genomes at the National Center for Biotechnology Information (NCBI) GenBank archive (accessed 17 January 2018). Genomic data paired with source metadata suggest that certain *Salmonella* lineages have preferred host ranges, and regulatory agencies such as the Food and Drug Administration (FDA) are exploring this aspect of *Salmonella* biology in the hope of facilitating rapid source attribution and mitigation of foodborne illness [4].

*S. enterica* serovar Montevideo (hereafter Montevideo) is consistently on the Centers for Disease Control and Prevention's (CDC's) list of the 20 *Salmonella* serovars causing human illnesses [5]. Sources of Montevideo outbreaks vary, with recent cases attributed to black and red peppercorn [6, 7], tahini [8] and pistachios [9]. Previous studies have also established that Montevideo is frequently associated with healthy cattle and can be isolated from ground beef [10, 11], yet human salmonellosis outbreaks of Montevideo associated with ground beef contamination are extremely rare [12]. This disparity fuelled our interest in characterizing the genomic differences between Montevideo strains isolated from healthy cattle and beef products, and those isolated from human patients and outbreak sources.

Previous phylogenetic analysis of 47 Montevideo strains revealed that this serovar is composed of four distinct clades on the basis of SNPs present in the Montevideo core genome [9]. Despite the substantial amount of Montevideo sequence data generated in that study, there are few complete genomes of this serovar from either bovine or human sources available within public databases [13]. Consequently, there has been no comprehensive genome-wide analysis of differences between the Montevideo clades. To better understand the genomic features that distinguish Montevideo isolates in each of the four clades, we generated complete closed reference genome sequence data for 13 Montevideo isolates (five from cattle sources and eight from human sources) and performed a comparative analysis of *Salmonella* pathogenicity islands (SPIs), secreted effectors, prophage content, fimbrial operons and genomic islands (gene clusters that may enhance bacterial fitness and are likely to have been acquired by horizontal gene transfer) with other publicly available Montevideo sequences (*n*=72) [9, 14, 15]. To expand the genomic scope with regard to differences in genes, genetic synteny and possible impact on pathogenicity, we further assessed genomic similarities and differences of Montevideo strains in comparison with *S. enterica* serovar Typhimurium strains, as they are the best studied with regard to host–pathogen interactions and virulence mechanisms [16, 17]. Variation in genomic gatekeepers such as clustered regularly interspaced short palindromic repeats (CRISPRs) [18] and DNA modification and restriction modification (R-M) systems [19] was also examined. CRISPRs provide adaptive immunity to foreign genetic material such as bacteriophages [20] and R-M systems act as innate immunity in restriction of foreign DNA [21]. While the roles of these gatekeeper systems are not yet fully understood, our analyses suggest that differences in CRISPR-associated (*cas*) gene organization may impact CRISPR activity and, subsequently, genome plasticity. Overall our analyses revealed distinct differences in gene content among members of the four Montevideo clades, especially with regard to prophage distribution, and genes encoding secreted effectors and virulence factors. The possible impact of these differences on the evolution of Montevideo lineages, variation in virulence gene content and the potential influence on niche specialization will be discussed.

## Methods

### DNA isolation, sequencing and assembly

Thirteen Montevideo strains were isolated from human clinical cases or cattle sources (ground beef, hide or subiliac lymph nodes). Isolation date, location, source and *XbaI* PFGE pattern were evaluated for each isolate to ensure genetic diversity (Table S1). Antimicrobial susceptibility phenotypes were determined by broth microdilution (CMV2AGNF, Sensititre, Trek Diagnostics, Thermo, Fisher) using Clinical and Laboratory Standards Institute (CLSI) minimum inhibitory concentration (MIC) breakpoints. The antimicrobial agents tested in this platform are listed in Table S1. Two of the genomes, USMARC-1903 and CDC 86-0391 (formerly USMARC-1921), have been previously submitted to NCBI GenBank [13]. Static bacterial cultures were grown at 37 °C in trypticase soy broth (Becton, Dickinson) overnight and DNA was isolated using Genomic-tip 100/G columns following the manufacturer’s protocol (Qiagen). Sequencing libraries were prepared according to Pacific Biosciences’ recommended protocol for P4-C2/P5-C3 chemistries. Single-molecule real-time (SMRT) sequencing was performed using a PacBio RS II instrument (Pacific Biosciences) to a mean coverage of 164× for the 13 isolates. Hierarchical genome-assembly process (HGAP v3.0) was utilized within the PacBio SMRT analysis pipeline to generate initial chromosome and plasmid unitigs. Unitigs were trimmed to remove putative redundant sequence from one or both ends of the unitigs, followed by circularization of the unitig in Geneious 10.1.3 (Biomatters). The program Ori-Finder [22] was used to identify the origin of replication in the trimmed, circularized unitigs, and the predicted origin was then reset as the first base of a re-linearized version of the chromosome/plasmid. The linear chromosome/plasmid sequence was then imported and subjected to the Resequencing protocol in SMRTportal v2.3. This step both provided an additional polishing of the assembly and served to validate the trimming and circularization steps by examination of reads mapping across the spot where the original circularization was performed. The complete Montevideo genomes were annotated by the NCBI Prokaryotic Genome Annotation Pipeline (PGAP) [23] and nucleotide sequences and statistics were submitted to GenBank (accession numbers listed in Table S1). The RS_Modification Motif_Analysis.1 protocol from the smrtanalysis v2.3 with the default threshold quality value (QV) of 30 was used to identify DNA base modification. Corresponding motifs of DNA methylases were identified by REBASE [24]. The PacBio base modification summary files were deposited in GenBank.

### Phylogeny and comparative genomics

The 13 Montevideo strains (Table S1), other publicly available Montevideo genomes and Montevideo strains from Allard *et al.* [9] were used for phylogenetic reconstruction through the Parsnp program from the Harvest suite of core-genome alignment and visualization tools [25]. In total, 85 Montevideo genomes were used (Table S1), although only 14 were complete and closed, including the 13 sequenced and analysed in this study. The sequences generated in this study also were uploaded to the EnteroBase website [26] and analysed to determine sequence type (ST) and eBurst group (eBG) based on SNPs within seven housekeeping genes (referred to as ‘legacy MLST’ [26]). The EnteroBase site was further queried for Montevideo sequence data entries and the resulting sequences (*n*=2204) were used to develop a minimal spanning tree (MST) using MSTreeV2, describing the relationship between the four major Montevideo clades. Metadata from EnteroBase were used to colour members of a given ST by source type. For Parsnp, the PhiPack option (‘-x’) for recombination and forcing of collinear regions (‘-C 1000’) were used, with CDC 86-0391 used as the reference genome. The phylogenetic tree was used as a guide for whole-genome alignment of the 13 Montevideo strains sorted by clades using Mauve [27, 28]. Mauve revealed the 13 genomes are highly collinear with no extensive chromosomal rearrangements of non-phage DNA. The Mauve alignment was used to identify chromosomal regions that consistently differed among the clades, and EasyFig blast n [29] was used for visual comparison of a number of key regions of difference. Geneious 10.1.3 was used to construct and query a custom *Salmonella* blast (blast v2.6.0) database derived from the Virulence Factor Database [1] to identify secreted effectors and fimbrial genes within each strain sequenced in this study. Default Megablast settings were run with linear gap penalty, scoring match mismatches of 1–2, an E-value cutoff of 1e-100 and word sizes of 28. For the majority of the virulence gene sequences, the Typhimurium LT2 genome was used as the reference for calculating relative pairwise nucleotide identity. Reference sequences for virulence genes not found in Typhimurium LT2 but identified in other *Salmonella* serovars or bacterial genera are noted accordingly.

PHASTER [30] was used to identify putative integrated prophages present in the genomes. After the initial identification of these prophages by PHASTER and insertion sites by REPFIND [31], Mauve alignments and manual inspection of prophages to known phages were used to confirm PHASTER calls. These were then designated as phage regions and are labelled as SM Φ1, Φ2, etc., for pangenome analysis. Pangenome analysis of the 13 Montevideo strains sequenced involved manual construction of a synthetic Montevideo pangenome by concatenating accessory metabolic island and phage regions into respective sites. The resulting 5.35 Mb sequence was set as a reference genome in GView Server [32, 33]. The genome sequences were ordered in the GView Server using the Montevideo phylogenetic tree as a guide. USMARC-1904 was set as the innermost ring and all genomes were compared to the constructed reference genome with blast n (E-value cutoff=1e-10, alignment length cutoff=100, percent identity cutoff=80). Gaps in the resulting output, indicating the location and distribution of prophages and other genomic islands, were coloured and labelled accordingly.

CRISPR spacers were identified by CRISPRdetect [34] using default settings (word size=11, min. word repetition ≥2, max. distance between words=125, min. repeat length ≥11, min. number of repeats ≥3, and CRISPR likelihood score ≥4). CRISPRtarget [35] was used to identify potential bacteriophage and plasmid sequences targeted by spacers in the CRISPR arrays, using default blast screen parameters (gap open=−10, extend=−2, nucleotide match=+1, mismatch=−1, E-value cutoff=1, word size=7, score ≥20). CRISPRTarget databases include Genbank-Phage, Refseq-Plasmid and Refseq-Viral. CRISPR spacers were visualized with the CRISPR DB II Excel Macro developed by Dr Philippe Horvath of DuPont [20] and organized as previously described [36].

## Results

The genomic data analysed in this study included both complete finished genomes generated using single-molecule long read technologies, and publicly available partial genome assemblies created from short reads (accession numbers in Table S1). The analyses include construction of a phylogenetic tree representing all 85 isolates with sequence data, examination of the 13 finished genomes for mobile element (e.g. prophage) content and location, genomic islands, comparison of immune defence loci, and identification of DNA base modification genes and their effects. To investigate the nature of variation within the Montevideo serovar, we analysed its key genomic features as described in detail below. Furthermore, any unique features identified within representatives of a given clade were compared against all Montevideo strains in the phylogeny (*n*=85 strains) using blast n analysis, in order to examine if the element was a common feature of that clade. Accordingly, unless noted otherwise, all metabolic islands, pathogenicity islands and genomic features examined in this study represent, to the best of our knowledge, conserved elements within a given clade. Antibiotic susceptibility to 15 drugs tested in the Sensititre CMV2AGNF panel was determined and all strains were found to be pansusceptible (Table S1).

### Phylogeny

The 13 Montevideo strains (including two previously reported [13]) were combined with one other previously finished genome (strain 507440-20; [9]) and 71 publicly available partial assemblies (Table S1), for a total of 85 strains, to generate a whole genome-based phylogeny tree using Parsnp [25] (Fig. 1a). The CDC 86-0391 genome was selected as the reference genome because it was free of prophages, and therefore provides clearer relationships among strains. Isolates within each clade were highly clonal, as indicated by the short branch lengths in the phylogenetic tree. The results presented here demonstrate that the 13 finished genomes sequenced for this study are distributed across all four clades, with seven isolates in clade I and two each in clades II–IV, respectively. Multilocus sequence type (MLST) analysis of the strains sequenced in this study using EnteroBase revealed that clade I isolates were ST138 and members of eBG 39, while clades II, III and IV were ST4, ST81 and ST316, respectively, and members of eBG 40. Examination of the source type metadata for 2204 Montevideo strains in EnteroBase supported previous suggestions of lineage niche association [9], as isolates in clade I appear to be predominantly associated with cattle sources (51 % of 951 strains), those in clade II with humans and poultry (17.9 and 17 % of 435 strains, respectively), clade III with water and humans (32.6 and 8.7 % of 377 strains, respectively), and clade IV, previously described as the Montevideo ‘outbreak’ clade [9], contained isolates from plants, soil/dust, avian, and human sources (12.9, 12.9, 11.1 and 11.1 % of 441 strains, respectively) (Fig. 1b). As previously observed, the majority of publicly available Montevideo genomes in GenBank are clustered within clade IV and it is noteworthy that many of the isolates in this clade have been linked to contaminated plant products including pistachios, tahini, sprouts and spices.

**Fig. 2.**
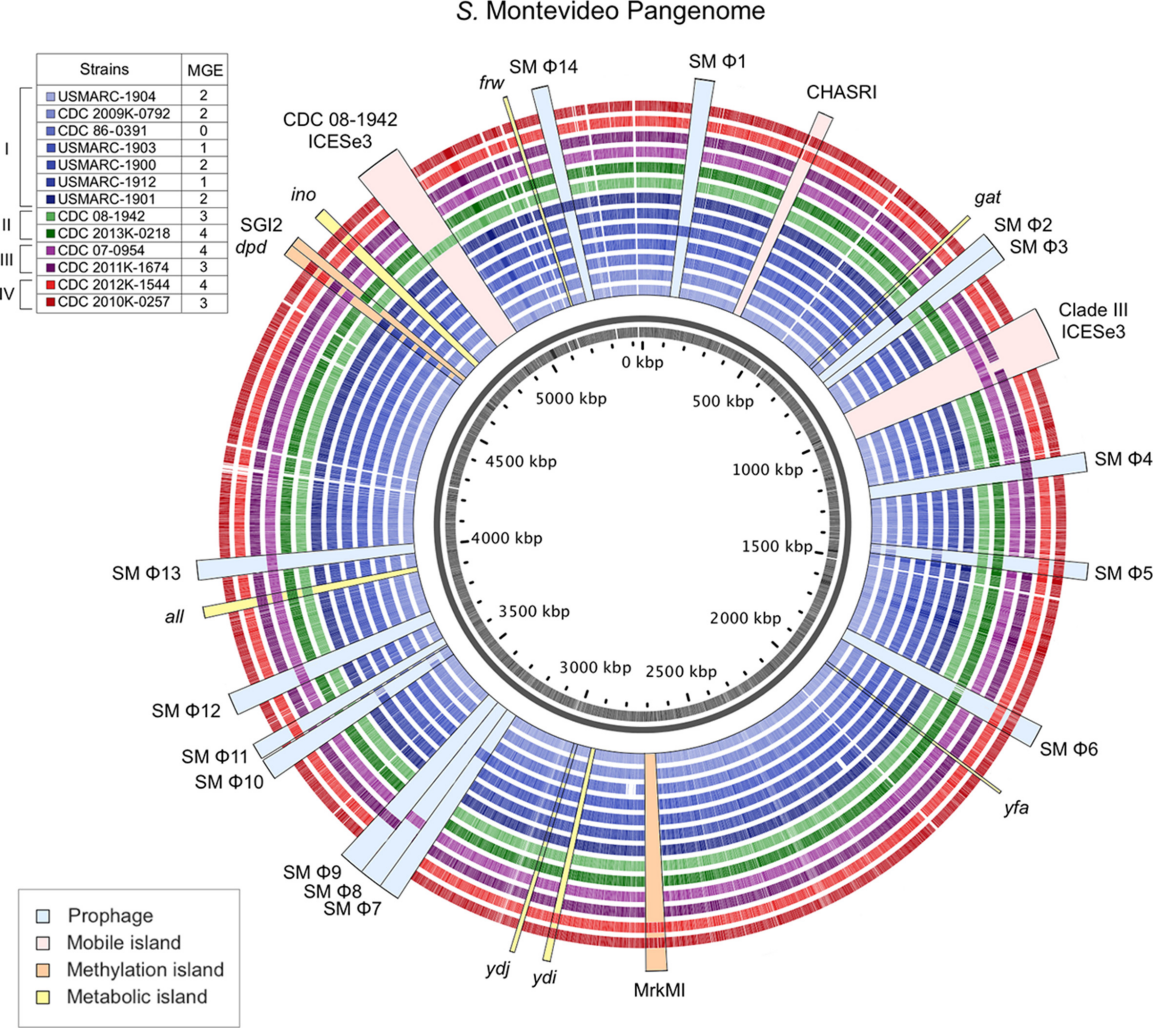
Summary of the pangenome of Montevideo strains sequenced in this study. A reference Montevideo pangenome containing all identified prophages, genomic islands and metabolic islands was constructed and was used as a blast reference for the 13 sequenced isolates sorted by clades. SM Φ1–14 are prophage regions identified by PHASTER and metabolic islands are defined in Fig. 1. MGE, prophages and mobile islands.

Comparisons of the genomes of 13 Montevideo isolates showed no evidence of large-scale duplications or inversions, although pangenome analysis sorted by clades revealed distinct regions of variation (Fig. 2) that included differences in mobile genetic elements (MGEs), effectors/virulence factors, fimbrial operon content, and methylated DNA motifs (summarized in Tables 1–3, and S1). We analysed these regions for potential impact on niche association and virulence, focusing on factors potentially affecting the outbreak clade occupancy.

**Table 1.** Percent nucleotide identity of identified effectors and virulence factors Typhimurium LT2 was used as a reference strain except where indicated by parentheses. Percent identity is colour-coded based on similarity distances. *sopE was only present in SM Φ7 in USMARC-1903.

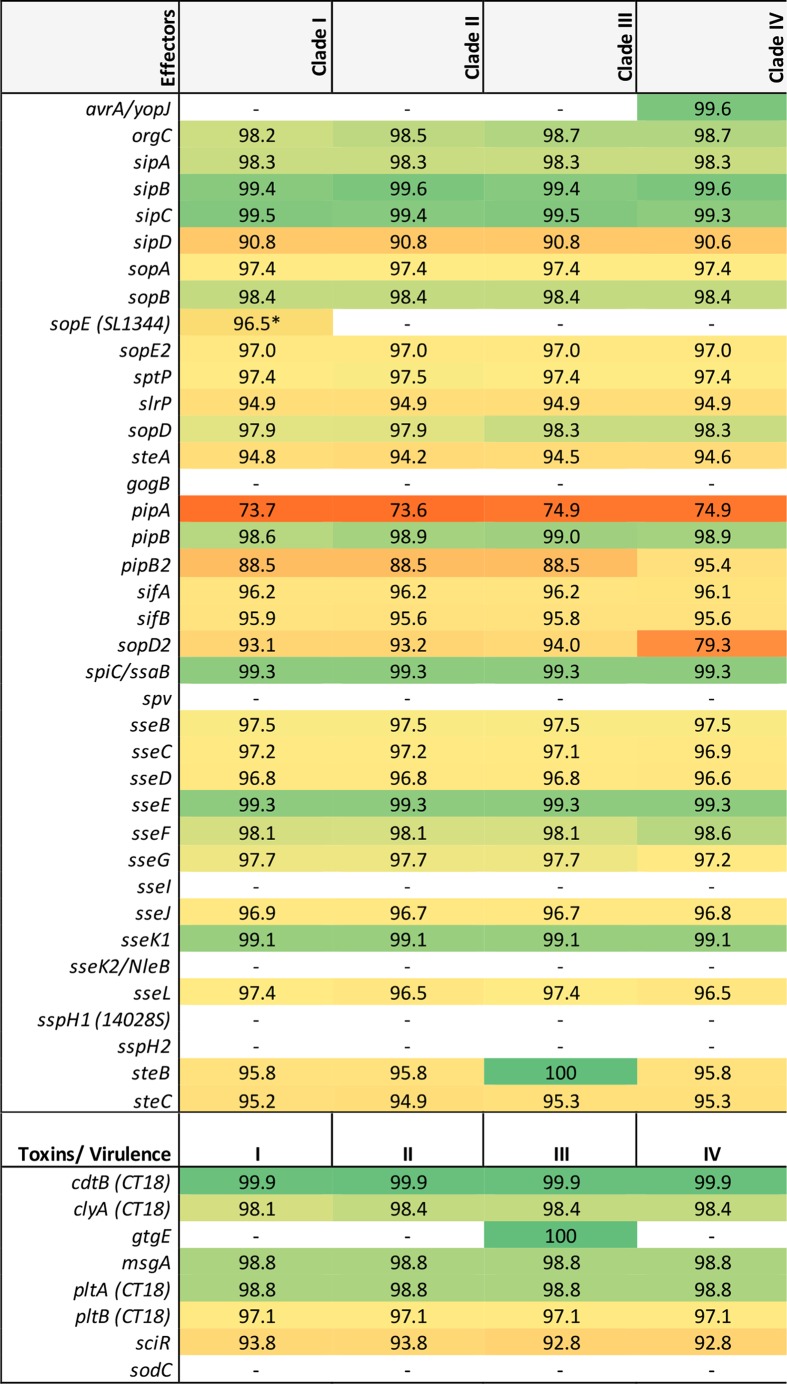

**Table 2.** Percent nucleotide identity of identified fimbrial genes Percent identity is colour-coded based on similarity distances.

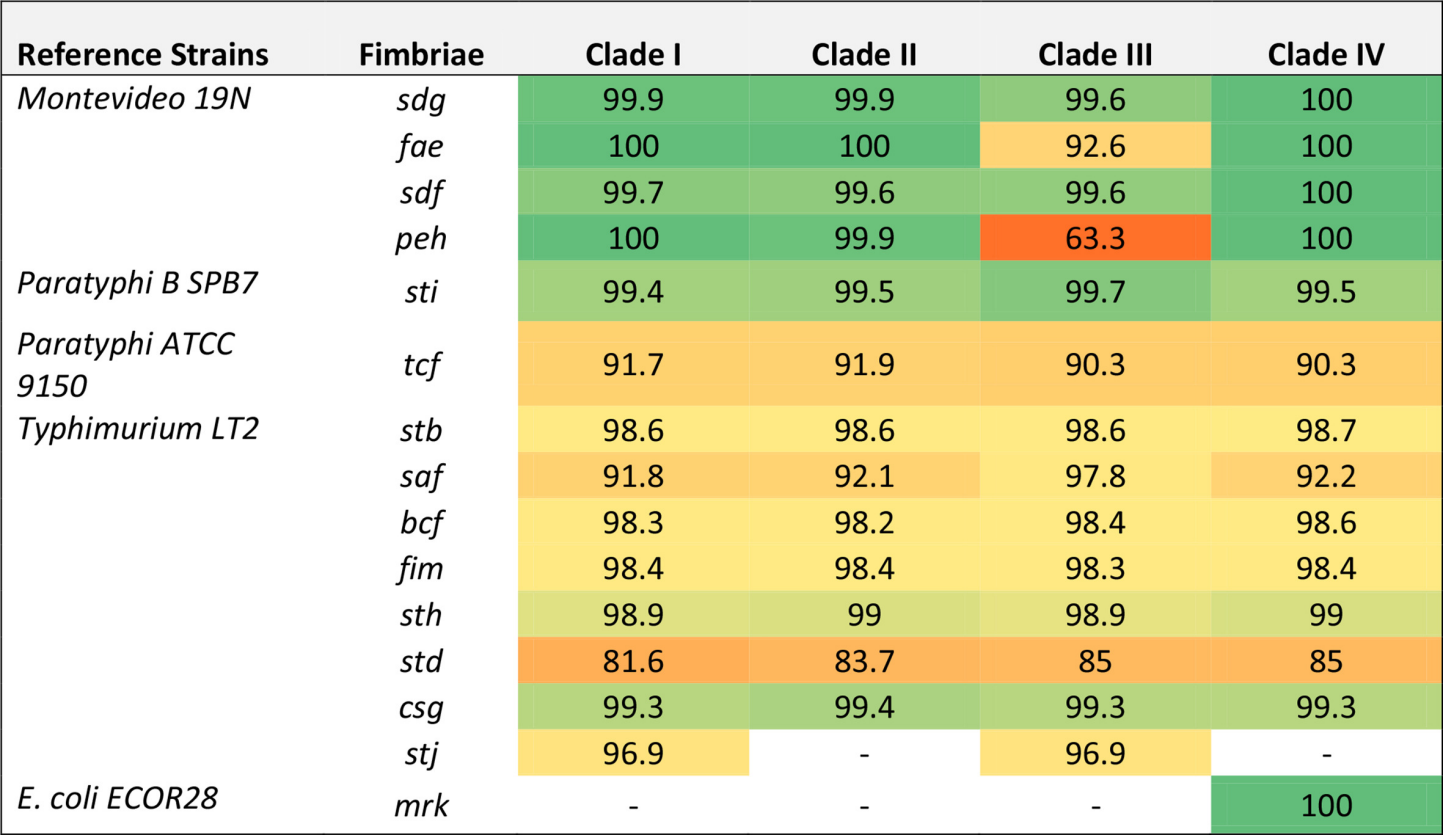

**Table 3.** Methylated DNA motifs detected in this study Motifs are listed if DNA modification was detected and if the cognate enzyme was identified by ReBASE. An underscore indicates the base that is methylated on the opposite strand. Core motifs are unshaded while partially conserved are shaded in red. Motifs shaded in yellow indicate the associated R-M systems are encoded on genomic islands (name in parentheses).

	G^m6^ATC	ATGC^m6^AT	CAG^m6^AG	CG^m6^AYN_7_RTRTC	CG^m6^AYN_7_ATGC	GNNT^m6^AYN_5_RTGG (SGI2)	TC^m6^AGN_6_TGC (MrkMI)
Clade I	×	×	×	×			
Clade II	×	×	×		×	×	
Clade III	×	×	×	×			
Clade IV	×	×	×	×			×

### Prophage differences

PHASTER analysis [30] examining phage content of the 13 Montevideo genomes sequenced revealed consistent differences among the four clades. Strains in clade I were observed to have the smallest genomes (ranging from 4.48 to 4.59 Mb) and were on average 139 kb smaller when compared to clades II–IV (Table S1). Much of the difference in average genome size among the clades can be attributed to horizontal gene transfer from bacteriophages and genomic islands as revealed by the pangenome analysis (Fig. 2). Prophage sequences identified in the genomes were classified by their closest matches using PHASTER analysis, with the top three scores shown in Table S2, to support assignment of putative designations (genomic regions designated as SMΦ1–14; Fig. 2). Questionable or incomplete PHASTER calls were visually examined and further inspected using blast analysis to make the most likely designation, and all prophage calls are summarized in Table S2. A Montevideo pangenome containing all accessory genomic island and phage regions of the 13 strains sequenced was manually constructed as described in Methods and the phage regions were then labelled in order of occurrence as SMΦ1–14 (Fig. 2).

Clade I strains were found to contain two or fewer large (~30–60 kb) integrated MGEs, while clades II–IV all have at least three and as many as four (Fig. 2). The isolate with the smallest genome sequenced in this study (CDC 86-0139; 4.48 Mb) has no recognizable putative intact phage regions, and is further reduced in relative size by a 33.6 kb deletion. This deleted region includes genes involved in at least three putatively identifiable activities, including an osmoprotectant system (*osmU*) previously identified in *Salmonella* [37], the *marRAB* operon that has been shown to be instrumental in resistance to multiple antibiotics and disinfectants [38], and *ydeJ* which encodes a protein with a putative competence/damage-inducible protein A domain [39].

A phage attachment site (i.e. genomic insertion site) targeted by the lambdoid prophage Gifsy-2, noted for contributing to virulence in Typhimurium LT2 (which integrates between *pepN* and *pcnB*), was occupied by various prophages in Montevideo clades I and III (SM Φ7-Φ9). A Gifsy-1/Gifsy-2/SEN34-like prophage (SM Φ8-Φ9) containing the virulence factors *gtgE* and *msgA*, but lacking *sodCI*, was present in all strains of clade III with the exception of ATCC BAA710. Another prophage (location SM Φ7) in USMARC-1903 is integrated in the same insertion site and is related to the SfI, SfV and SEN34 prophages. This phage appears to be distantly related to Gifsy-2 with a similar integrase (data not shown) but does not carry homologues to *gtgE*, *msgA or sodCI*. Rather, it contains an ORF with 96.5 % homology to the prophage-encoded virulence factor *sopE* in the bovine isolate Typhimurium strain SL1344 [40, 41]. The other prophages described in this study (occupying regions SM Φ1–6 and SM Φ10–14) do not encode any identified virulence factors and it is unknown what impact these prophages impart on bacterial fitness.

Clade IV is particularly interesting with regard to the number of putatively horizontally acquired elements (Fig. 2). The profile of prophages found in isolates from this clade is consistent with the previously reported clonal nature of its members [8]. Virtually all clade IV isolates display the Fels-2-like and Salmon_vB_SosS_Oslo-like prophages (SM Φ5 and SM Φ6, respectively), although they are not unique to clade IV (Fig. 2). They also share two genomic islands including the copper homeostasis and silver resistance island (CHASRI; 32.4 kb) [42] and a 40.8 kb R-M island carrying a unique methyltransferase, integrase and the fimbrial operon, *mrk*, previously observed in *Klebsiella* and *Citrobacter* [43] and here designated MrkMI (for Mrk , Methylation island) in Fig. 2.

### ICE and metabolic island differences

An integrative and conjugative element (ICE) designated ICESe3 has been previously described in *S. enterica* [44] and has similarity with SPI-7 (Fig. S1). This SPI-7-like ICESe3 element (76.3–107.9 kb) was identified in three of the Montevideo isolates for which we generated finished genome sequences, including one from clade II (CDC 08-1092) and two from clade III (Fig. 2). Comparison of the ICESe3 element sequence against all the sequences in the Montevideo phylogeny using blast n analysis indicates that this element is a common feature of all clade III isolates, with the exception of ATCC BAA710. The ICESe3 element is highly conserved among the three finished genomes, sharing modules for conjugative transfer, the type IVB pili and haemagglutinin afimbrial adhesins (Fig. S1), supporting a hypothesis of horizontal transfer among clades. The ICESe3 in CDC 07-0954 has an insertion of a duplicated module that contains an extra haemagglutinin afimbrial adhesin, accounting for size differences between the elements. However, the Montevideo ICESe3 do not encode the *sdi* or *sbb* fimbrial genes associated with the canonical ICESe3 (accession FN298495) [45].

Additional small horizontally acquired elements involving metabolic genes were identified, including an arsenic resistance island that was found present in all clades of Montevideo [46]. In addition, a tagatose utilization island *tag*, which spans STM3251 to STM3256 in Typhimurium [47], was found but restricted to members of clades I and II (Fig. 2). Clades I and II are also distinguished by the presence of an l-rhamnonate metabolism islet, *yfa* [48], that is present in many other NTS serovars, and the putative major facilitator transport system island *ydj* [49, 50] (Fig. 2). The allantoin metabolism island (*all*), which is in all Typhimurium isolates except for the DT104 lineage [51], and has also been found in *Klebsiella pneumonia* isolates associated with liver abscesses in humans [52], was found to be present in Montevideo but restricted to isolates from clades II and III (Fig. 2). The fructose-like phosphotransferase system island (*frw)* was found only in clade II [53] (Fig. 2). Clade IV strains were found to contain a 14.7 kb *ydi* island which may be involved in utilization of quinate/shikimate [54, 55], and a 23.1 kb *myo*-inositol (*ino)* metabolism island that has also been identified in Typhimurium [56] (Fig. 2). The ORFs corresponding with these metabolic island differences are further summarized in Table S3.

### Plasmid differences

Only one of the isolates sequenced in this study (CDC 2010K-0257) contained a plasmid (Table S1). This plasmid, designated pSMO-2010K-0257, is a type 1 IncA/C_2_ plasmid that is 119.8 kb in length and does not encode any identifiable antibiotic resistance genes. This plasmid does not encode any known plasmid-associated virulence factors such as the *spv* locus [57].

### *Salmonella* pathogenicity island (SPI) differences

Montevideo isolates across all four clades harbour SPIs with homology to SPI-1 to SPI-6, which have been described for other *Salmonella* serovars [58]. However, we observed variations in the sequence of SPI-1, -3, -5 and -6 as compared to the analogous SPIs found in other serovars. In SPI-1, the secreted effector encoded by *sipD* is present in Montevideo strains, but shares only ~88 % amino acid identity with the Typhimurium SipD (Table 1). In addition, we identified the gene encoding the secreted effector AvrA (also known as YopJ) in SPI-1 of all Montevideo clade IV strains (Table 1), despite previous reports that *avrA* was absent in SPI-1 of this serovar [59, 60]. For SPI-3, we did not observe *sugR* or *rhuM* in any Montevideo strain. The absence of these two genes in SPI-3 has also been previously observed for other *Salmonella* serovars [61]. In SPI-5, the protein encoded by *pipA* has a 53 amino acid deletion in all Montevideo clades, relative to that encoded by *pipA* in Typhimurium. A related virulence gene, *pipB2*, which is not encoded on an SPI, but is secreted by the SPI-2 type III secretion system [62] was observed to have an internal 20 amino acid deletion present in members of clades I, II and III, in comparison to LT2 (PipB2Δ176–195). This deleted region spans part of the pentapeptide repeat that is characteristic of the PipB family [62]. In contrast, the PipB2 in clade IV does not have the deletion and is the same size as the Typhimurium PipB2 at 350 amino acids, sharing 92 % amino acid identity.

**Fig. 3.**
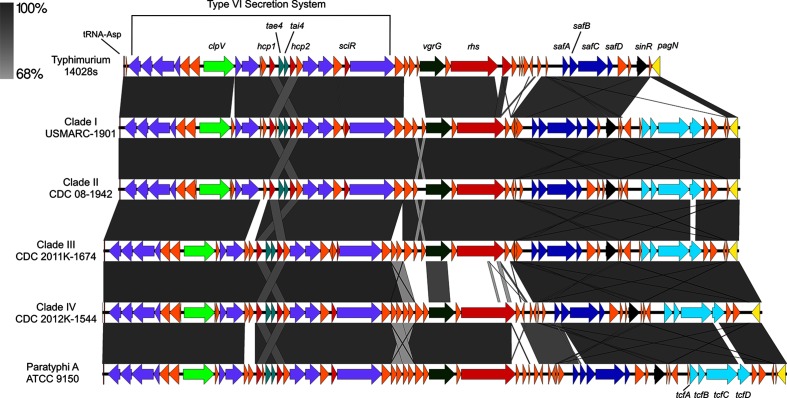
Conservation of SPI-6 of Montevideo clades and related *Salmonella* serovars. Type VI secretion system – violet, Clp ATPase *clpV* – light green, putative virulence factors (*hcp*, *sciR*, *rhs*) – red, phage-like tailspike *vgrG* – dark green, *saf* fimbrial – blue, *tcf* fimbriae – light blue, *sinR* transcriptional regulator – black, adhesin/invasin *pagN –* yellow.

SPI-6 was observed to have the most variability across Montevideo clades, and in comparison, with other *Salmonella* serovars (Fig. 3). The SPI-6 of all Montevideo examined has an additional fimbrial operon (*tcf*) which is lacking in Typhimurium, but is similar to the SPI-6 of *S. enterica* Paratyphi A (Fig. 3). In addition, the *rhs* encoded toxin and *vgrG* tip genes in clades I–III were more homologous to those found in Typhimurium [63], while those in clade IV were more closely related to the *rhs* and *vgrG* in Paratyphi A (Fig. 3).

Other major differences were observed with SPI-12, which is present in Typhimurium but absent in all Montevideo sequenced to date, and contains the gene encoding the effector SspH2 which is important for trafficking and maturation of the membrane-bound compartment that *Salmonella* reside in when internalized by macrophages (the *Salmonella* containing vesicle or SCV) [64]. In addition, in the genomic island CS54, the *shdA* gene encoding fibronectin binding protein, noted for its role as a Peyer’s patch colonization factor in mice [65], was found to differ substantially between Typhimurium LT2 and that present in Montevideo strains (2040 vs 1776 amino acids in length, respectively) with only 71 % overall amino acid identity.

### Secreted effector, toxin and fimbrial operon differences

A comparison of known secreted effectors and toxins in Montevideo clades with respect to known effectors in Typhimurium revealed some differences between serovars [1] as well as inter-clade differences within Montevideo. Notably, effector genes *gogB*, *sseI*, *sseK2*, *sspH1*, and as mentioned above, *sspH2* [66] and the phage-encoded periplasmic Cu,Zn superoxide dismutase *sodCI* [67] were absent in all Montevideo examined (Tables 1, S4 and S5). In contrast to Typhimurium, the cytolethal distending toxin encoded by *cdtB* and the *artAB* operon (also known as *pltAB*) are present in all Montevideo as previously reported [68, 69]. As mentioned earlier, the *gtgE* virulence gene, similar to that found in the Gifsy-2 prophage [70], is present in all clade III members with the exception of ATCC BAA710 (Tables 1 and S5). The clade IV *sopD2* encoded effector displayed substantial nucleotide differences, specifically amino acids 64–75 within the conserved membrane-targeting domain, with respect to that found in clades I–III and in Typhimurium [71] (data not shown).

Previous studies of genes encoding fimbriae have primarily included Montevideo strains from clade IV [45]. The comparative analysis presented here shows that Montevideo possess a diverse repertoire of fimbriae, sharing core fimbrial loci *bcf*, *fim*, *saf*, *stb*, *std*, *sth* and *sti* with most other *Salmonella* serovars ([45]; Tables 2 and S6). While most of these core fimbriae genes were found to share a high degree of homology with Typhimurium LT2, the *std* fimbriae showed substantial differences (Table 2). Moreover, none of the Montevideo strains were found to have the *lpf* or *stc* fimbriae which are present in many other serovars [72]. The *mrk* fimbriae have been reported in Montevideo [45], but our analysis revealed that it is only present in clade IV and is identical to a previously sequenced *E. coli mrk* operon [43]. In addition, the *stj* fimbrial gene cluster found in Typhimurium was not previously reported in Montevideo [45], but is present in clades I and III. In clade III, the *peh* operon has diverged considerably from other Montevideo clades (Table 2) and closely resembles the *peg* fimbriae from Paratyphi A instead (97.2 % nucleotide similarity). Clade III *faeG*, encoding the major subunit of the K88 fimbriae [73, 74], has diverged considerably in comparison to other Montevideo clades (Table 2). In addition, the *safA* of clade III shares higher nucleotide identity to the Typhimurium LT2 *safA* (94.4 %) as opposed to ~76 % nucleotide identity in the other Montevideo clades (data not shown).

### Bacterial immunity loci and DNA modification systems

**Fig. 4.**
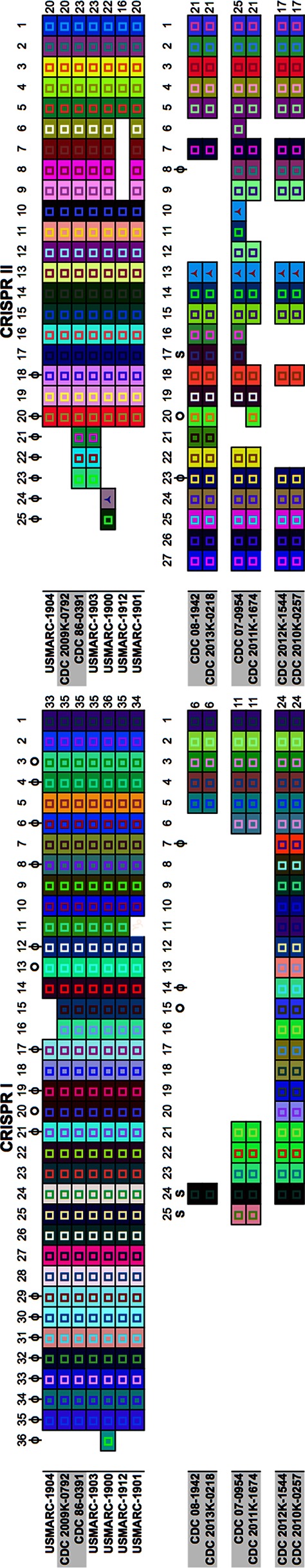
Montevideo CRISPR spacer arrays. Extended CRISPR spacer arrays in clade I and matches of these arrays to prophages suggests a more active CRISPR. The 'Φ' above the spacers indicates top hits against bacteriophages and/or plasmids while the 'ο' indicates hits against plasmids only. 'S' indicates self-hits and spacer tally is indicated on the right. Spacer sequences are converted into two colour symbols through the CRISPR DB II Macro. Spacers of the same colour and symbols indicate sequence homology.

The observed variations in Montevideo genome size and mobile element content led us to examine the structure and diversity of genomic gatekeepers, including CRISPR and R-M systems. Examination of the CRISPR spacer array revealed a striking difference between Montevideo clades (Fig. 4). Clade I Montevideo possess a greater number of CRISPR spacers (51–58) than other Montevideo clades (27–41), and an *in silico* search by CRISPRTarget [35] revealed an increased number of spacer sequences that target bacteriophage and plasmid sequences in clade I (Fig. 4). Notably, clade I spacers 29, 30, 31 and 34 in CRISPR1 and spacer 20 in CRISPR2 have 100 % nucleotide matches to sequences in vB_SosS_Oslo-like prophages (SM Φ6) identified in strains sequenced in this study (Fig. 4). Clade I spacers 21, 33 and 35 of CRISPR1 also target SM Φ6 prophages when allowing for up to three mismatches. Clade I spacer 12 of CRISPR1 has a 100 % match to the CDC 07-0954 prophage in SM Φ8. The CRISPR arrays in CRISPR2 are more similar in size among clades, although there are more spacers with matches against bacteriophages and plasmids within clade I. The clade I spacers 24 and 25 target both SM Φ8 and SM Φ9 prophages with a 100 % match and a 2 nt mismatch, respectively. The larger arrays in clade I negatively correlate with the number of mobile elements and average genome size in clade I when compared to the other Montevideo clades.

Three spacers in clades II–IV were found to be self-targeting (Fig. 4). Spacer 24 in CRISPR1 (clades II–IV) targets the anion permease *citT* gene with a single mismatch. Spacer 25 in CRISPR1 (clade III) targets an intergenic region between tRNA-Arg and *fimW* with a 100 % nucleotide match while spacer 17 of CRISPR2 (clades II and III) also has a 100 % nucleotide match in *ybjD* that encodes an ATP-dependent endonuclease. No self-targeting spacers were identified in clade I.

**Fig. 5.**
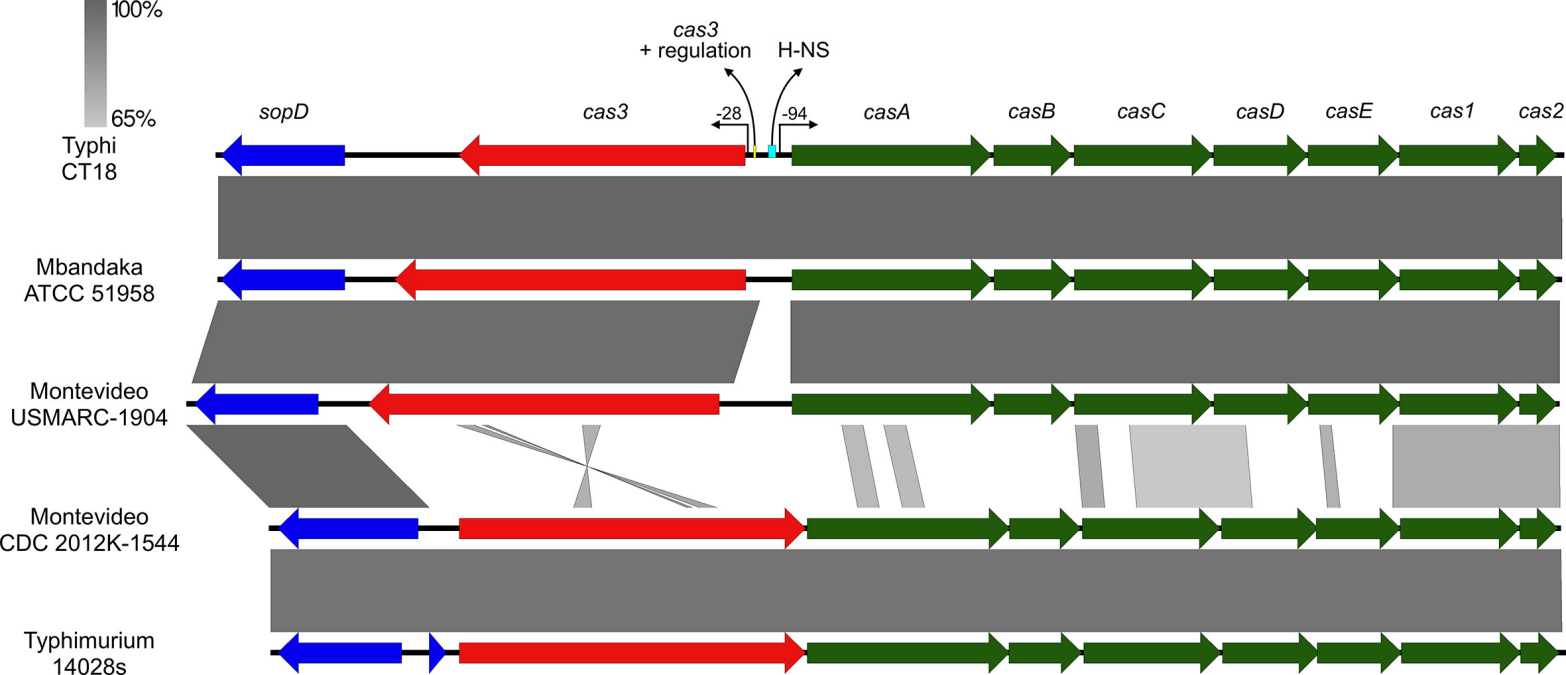
Divergent transcription of *cas3* in Typhi and clade I Montevideo. The *cas3* is divergently transcribed in Typhi, Mbandaka and in Montevideo USMARC-1904 (clade I) when compared to other Montevideo clades and Typhimurium. Regulation of *cas* is adapted from Medina-Aparicio *et al.* [75, 76]. Conserved host genes – blue, *cas3 –* red, other *cas* – green.

Alignment of the *cas* genes present in each of the Montevideo clades also revealed major differences. Clade I Montevideo has a divergently transcribed *cas3*, similar to the recently characterized *cas* in *S. enterica* Typhi (Fig. 5) [75, 76]. Unlike Typhi, however, the intergenic region between *cas3* and the *cas* operon is not shared between clade I Montevideo and the other divergently transcribed *cas3 Salmonella* serovars, suggesting different regulation of the *cas* genes in clade I, with respect to that in Typhi. In clades II–IV and most *Salmonella* serovars including Typhimurium, the *cas3* gene is transcribed as a single operon with other *cas* genes [36]. In addition to orientation and regulation, there are major differences in homology between *cas* genes in clade I and clades II–IV which may suggest different functionality or activity (Fig. 5). Comparison of homology of Montevideo *cas* to sequences in the NCBI database indicates that clades II, III and IV *cas* gene orientation is concordant with the majority of publicly available sequenced *Salmonella* genomes, including Typhimurium.

Differences in R-M systems among the clades were also examined because of their role in mediating genetic exchange and the resulting influence on bacterial lineage evolution. R-M systems encode enzymes that modify the nucleotide bases of specific sequence motifs that can either prevent or enhance cleavage by associated endonucleases. The kinetics of nucleotide incorporation during single molecule sequencing on the PacBio platform allows detection of DNA base modifications, particularly 6-methyl-adenosine (^m6^A) modification that is prevalent across bacterial species, and supports the comparison of modification profiles across isolates [77]. All Montevideo strains were observed to share three common core motifs displaying ^m6^A base modification (GATC, ATGCAT, CAGAG), while isolates from clades II and IV showed distinct differences in their methylation profile associated with additional motifs (Table 3). All motifs could be provisionally assigned to GenBank annotated methyltransferase enzymes using REBASE [24] prediction tools. The genes encoding the R-M systems, consisting of the DNA methyltransferase enzyme and associated endonuclease, are located in regions displaying variability between strains which are therefore tentatively termed ‘R-M cassettes’. The partially conserved modified DNA motif CG^m6^AYN_7_RTRTC (underscore indicates a modified adenine on the complementary strand and subscript indicate number of ‘N’ bases) and its cognate R-M cassette were detected in clades I, III and IV. The R-M cassette in the same genomic context in clade II recognizes CG^m6^AYN_7_ATGC instead and its cognate methyltransferase contains many SNPs in comparison to the cassette in clades I, III and IV. Clade IV possesses the aforementioned 40.8 kb MrkMI R-M cassette, integrated between an *S*-adenosylmethionine tRNA ribosyltransferase and an acyl-carrier protein gene, which encodes a Type I R-M system that recognizes TC^m6^AGN_6_TGC (Fig. 2). The MrkMI cassette also encodes an integrase, an additional and uncharacterized restriction endonuclease, haloacid dehalogenase, and the *mrk* fimbrial locus. A 7 bp direct repeat was observed flanking MrkMI (5′ ATTTAAC 3′), which may be a remnant or ‘scar’ of previous horizontal gene transfer [15].

The existence of a newly recognized form of bacterial DNA modification has also recently been reported in Montevideo [78], which results in the formation of 7-deazaguanine modifications. This modification is not presently detected by the PacBio base modification software, possibly because it is not common in bacteria and therefore the influence of the modification on sequencing kinetics is not yet established. However, the genomic locus responsible, containing tRNA guanosine transglycosylase (*tgtA5*) and 7-cyano-7-deazaguanine synthesis genes, has been described, and named a ‘*dpd* cluster’. This cluster integrates near tRNA-Leu [79], and has been reported to be present in ~92 % of sequenced Montevideo isolates [78]. In our analysis, the 7-deazaguanine *dpd* cluster was present in clades I, III and IV (Fig. 2), although we did not determine if it was actively modifying DNA in our strains. All clade II isolates lacked the *dpd* cluster, and instead carried an R-M cassette denoted SGI2 [79], which is putatively responsible for modification of the adenine in motif GNNT^m6^AYN_5_RTGG (Table 3). A 17 bp direct repeat (5′ CGAAGGCCGGACTCGAA 3′) was found flanking the clade II SGI2.

## Discussion

Here we describe the genetic differences that distinguish members of the four clades of *Salmonella enterica* serovar Montevideo. The complete closed reference genome sequence data of 13 Montevideo isolates generated in this study represent an important contribution to the broader goal of using whole genome sequencing for rapid source attribution, because prior to our submission, only three complete Montevideo genome sequences were available in GenBank. Comparative analyses showed that differences in prophage and metabolic island content were the major features delineating the four clades, and that distinct CRISPR-Cas systems may be the root cause for these observed differences.

Phylogenetic analysis of the sequence data generated from 13 strains in our collection, along with 72 publicly available genome assemblies (one complete and 71 draft) in GenBank, resulted in a tree with a topology that is highly congruent with the GARLI SNP-based phylogeny generated by Allard et al., using short read sequencing technology [9]. However, while the majority of strains analysed in Allard’s study were found to reside in clades II, III and IV, the majority of strains sequenced in our study were observed to group with clade I (seven of 13 isolates, five cattle-associated and two human; Fig. 1a). Given that a major goal of this study was to understand the genetic differences between Montevideo isolated from healthy cattle or beef products, and those isolated from infected humans, it is noteworthy that all cattle isolates in this study were found to group within clade I. In addition, the single clade I isolate analysed in Allard’s study was stated to have greatest similarity to isolates from pet treats made from beef [9]. This evidence, in conjunction with the source type attribution metadata for 2204 Montevideo strains present in EnteroBase, suggests that clade I Montevideo are predominantly bovine-associated (Fig. 1a, b).

Pangenome analyses revealed that genomes of clade I members were substantially smaller (by on average 139 kb) than those of the other clades and that a paucity of phage and genomic islands in clade I was the reason for this difference (Fig. 2). This observation led us to examine differences in the genomic gatekeeper systems, including the CRISPR-Cas and R-M systems, and revealed that CRISPR-Cas differed substantially among the clades. Specifically, CRISPR spacer analysis showed that arrays of clade I members were larger than those of the other clades, especially with regard to CRISPR I (Fig. 4). It has been shown previously that there are considerable differences in CRISPR spacer content both within and between various *Salmonella* serovars [36]. However, a novel discovery made here was that the Cas operon of Montevideo clade I members is very different from that in clades II–IV (Fig. 5). In clade I, the *cas3* gene is in the opposite orientation from the other genes in the Cas operon, while in clades II–IV (as well as in Typhimurium and most *Salmonella* serovars) *cas3* is in the same orientation as *casA–cas2* [36]. Moreover, examination of nucleotide identity of the clade I Cas operon genes with those of other *Salmonella* shows clade I to have greatest homology with the Cas genes of Typhi and Newport linage II (93.8 and 93.9% identity, as opposed to 54 % identity with the Cas in Typhimurium LT2), while clades II–IV have highest nucleotide identity with Typhimurium (94 %).

Accordingly, Montevideo clade I appears to be part of a short but growing list of *Salmonella* identified with this version of Cas gene orientation. The significance of this is not yet known but it is noteworthy that strains containing this Cas gene orientation appear to have longer CRISPR arrays, suggesting that this version may be more active. In support of this hypothesis, *S. enterica* Mbandaka, which also has a divergently transcribed *cas3* (Fig. 5) [80], possesses an enormous CRISPR array of 221 spacers [75, 76]. Growing evidence suggests that CRISPR-mediated immunity plays a crucial role in pathogen evolution by impacting the ability to acquire virulence determinants from the mobilome and/or by genome reduction from loss of metabolic genes [81]. As such, the unusual CRISPR system resident in clade I strains may have played an integral role in the observed niche association of this clade, by preventing the integration of phage-encoded pathogenicity-related gene functions.

As human pathogens, Montevideo serotypes are consistently ranked in the CDC’s list of top 20 *Salmonella* serotypes attributed to human illness, causing an average of 700 illnesses each year in the USA [5] (approximately 1–2 % of the total reported cases of salmonellosis). This is a fraction, however, of the illnesses attributed to the two top ranked serovars, Enteriditis and Typhimurium, which collectively account for approximately 30 % of salmonellosis cases each year (~20 and 10 %, respectively), and suggests that Montevideo strains may be less successful as human pathogens in comparison with the top ranked serovars. Within serovar Montevideo, it is noteworthy that strains from all four clades have been isolated from humans, although sequence data to date show the majority of strains associated with human illness or outbreak sources are members of clades II, III and IV, with the lion’s share of these residing in clade IV (Fig. 1a). While it is possible that the high relative representation of clade IV strains in human disease cases may be an artefact of ascertainment bias, or a result only of higher exposure to bacteria occupying this clade, it is worthwhile to consider the distribution of known determinants of pathogenicity among the clades. To that end we assessed the genomic similarities and differences of Montevideo strains in comparison with the highly successful human pathogen, Typhimurium. This analysis revealed that with a few exceptions noted in Table 1, Montevideo generally lack a number of prophage encoded virulence factors that have been shown to enhance Typhimurium pathogenicity, including the Gifsy-1 encoded *gogA* and *gogB*, Gifsy-2 encoded *sseI*, *gtgA* and *gtgE*, the Gifsy-3 encoded *sspH1,* the SopEϕ encoded *sopE*, and the Fels-1 encoded *sodC1* (Tables 1, S4 and S5). These factors play important roles in *Salmonella* survival and persistence within host cells by down-regulating the host immune response or protecting *Salmonella* from host defences [82]. Other virulence genes that are generally not present in Montevideo strains include the plasmid encoded *spv* virulence genes (shown to function in delaying epithelial cell death), the *sseK2* encoded glycosyl transferase (noted for inhibiting host antibacterial and inflammatory response) and the SPI-12 encoded *sspH2* (shown to be important in trafficking and maturation of the SCV). The picture that emerges from this comparative analysis is that Montevideo strains may be less successful as human pathogens because they appear to have fewer mechanisms for modulating the host immune system, avoiding clearance and surviving host defences within the SCV.

Taking these comparisons a step further to define genomic differences among the four clades of Montevideo revealed distinct patterns of metabolic island and virulence gene content. Specifically, clades I and II were found to contain metabolic islands noted for conferring the ability to transport and utilize tagatose (*tag*) and l-rhamnoate (*yfa*), as well as an aldo/keto sugar kinase (*ydj*), that were lacking in clades III and IV (Table S3). Clade II strains also were observed to contain islands putatively conferring the ability to utilize allantoin and a fructose-like phosphotransferase system. Given the apparent niche association of clade I isolates for cattle, and clade II isolates for poultry, it is possible that these metabolic islands contribute to bacterial colonization in livestock. In support of this suggestion, it has been shown that Typhimurium *tag* operon mutants were markedly attenuated in their ability to colonize the intestines of cattle, swine and poultry [47, 83], and in serovar Enteritidis, allantoin utilization genes are upregulated in chicken intestines [84, 85], while *allB* deletion mutants demonstrate reduced virulence in poultry [51]. However, further research is needed to understand how these metabolic capabilities actually impact fitness in colonizing livestock.

Clade III strains were unique among the Montevideo strains characterized in that all representatives sequenced to date and present in GenBank have been isolated from humans (Fig. 1a). Analysis of source type metadata for clade III (ST81) representatives in EnteroBase showed water (32.6 %) and humans (8.7 %) to be the primary sources for members of this lineage (Fig. 1b). The defining genomic features identified in clade III included an ICESe3 element reminiscent of SPI-7 in Typhi (encoding a haemagglutinin afimbrial adhesin and type IVB pili), a Gifsy-like prophage encoding the secreted effector *gtgE* (noted for interfering with bacterial clearance by preventing SCV from fusing with host lysosomes), and a distinct repertoire of fimbrial gene clusters (Fig. S1 and Tables 1 and 2). Overall, these elements suggest that clade III has evolved within a distinct niche (or niches) requiring different attachment mechanisms than those present in the other Montevideo clades.

The most striking genomic differences catalogued in this study, however, were found in the ‘outbreak’ clade, clade IV. These included genes implicated in bacterial colonization of vegetables, nuts and seeds, as well as survival in soil [i.e. the heavy metal resistance island CHASRI [86], the methyltransferase and *mrk* fimbrial operon (here noted as MrkMI) [45, 87, 88], the *myo-*inositol (*ino*) metabolism island [56, 89] previously characterized in Typhimurium [56], and the quinate/shikimate (*ydi*) utilization island (Fig. 2 and Table S3)]. Specifically, CHASRI has been reported to be widespread in bacteria isolated from fresh produce [86] while *mrk* fimbriae have been shown to be important for bacterial adhesion to plant roots [87, 88]. However, the presence of the *myo*-inositol metabolic island provides the most compelling evidence for a genetic mechanism underlying clade IV isolates being plant-associated, given that seeds and nuts (common vehicles for Montevideo outbreaks) contain concentrated levels of *myo*-inositol, which is noted for its role in energy and phosphorus storage in seeds and plants [90]. Moreover, clade IV members were found to encode virulence factors including the secreted effector AvrA, noted for dampening host immunity [60, 91, 92], and a Typhimurium-like PipB2 shown to be important in SCV maturation and maintenance (Table 1), as well as a version of SPI-6 showing greater homology with that found in serovar Paratyphi A than Typhimurium (Fig. 3). Taken together, these elements suggest that the success of Montevideo clade IV strains as human pathogens may be attributed to a combination of fitness advantages for colonizing and persisting within food types that are not normally cooked, and virulence traits that aid in modulating the host immune system and avoiding clearance. Our identification of Montevideo clade IV strains carrying these traits may facilitate the development of screening tools specific for this clade, which could have important implications for public health.

In conclusion, we document here numerous details of prophage, genomic island, SPI, effector/toxin/fimbrial gene content, CRISPR-Cas and R-M system variation among and within clades of Montevideo. In a previous phylogenetic analysis of Montevideo, Allard *et al*. concluded that members of this serovar appear to be monophyletic in nature [9]. MLST analysis of the Montevideo strains sequenced here, as well as 2204 Montevideo strains present in EnteroBase, reveals that clade I strains (ST138) are members of eBG 39, while clades II (ST4), III (ST81) and IV (ST316) are members of eBG 40. That Montevideo strains can be found in two different eBG raises the question of whether they are polyphyletic or truly monophyletic. However, genomic comparisons of members of the four clades reveal the presence of a number of features shared by all members of this serovar, including the arsenic resistance island, genes encoding the cytolethal distending toxin (*cdtAB*), the lack of SPI-12 and the presence of an additional fimbrial operon (*tcf*) in SPI-6. These common features in addition to the overall gene synteny observed across the Montevideo genomic landscape provide support for the hypothesis that Montevideo is monophyletic, but that the clades diverged early in the evolution of this serovar, and subsequently may have adapted independently to different environmental niches. It is evident that horizontal gene transfer and gene loss have played a marked role in the diversification and apparent niche association of Montevideo. The data presented here shed light on the drivers that shape *Salmonella* genome plasticity and probably influence the propensity for acquiring virulence and fitness factors that impact success in a given niche. Moreover, they provide a framework for future studies aimed at characterizing the impact of these drivers on *Salmonella* serovar and sublineage evolution.

## Data bibliography

1. Tatusova T, Ciufo S, Fedorov B, O'Neill K, Tolstoy I. Refseq Prokaryotic Genome Annotation Project. doi: 10.1093/nar/gkt1274 (2015).
2. Kong N, Davis M, Arabyan N, Thao K, Ng W, Huang BC, Chen P, Weis AM, Chin N, Foutouhi S, Foutouhi A, Storey D, Xie Y, Kaufman J and Weimer BC. 100K Foodborne Pathogen Genome Project at University of California at Davis PRJNA203445 (2016).
3. McDermott P, Zhao S, Li C, Tyson G and Lam C. *Salmonella enterica* MDR Genome sequencing. PRJNA242614 (2015).
4. Timme R, Allard MW, Strain E, Evans PS and Brown E. Whole genome shotgun sequencing of cultured foodborne pathogen. PRJNA161681 (2014).
5. Timme RE, Pettengill JB, Allard MW, Strain E, Barrangou R, Wehnes C, Van Kessel JS, Karns JS, Musser SM and Brown EW. Phylogenetic Diversity of the Enteric Pathogen *Salmonella enterica* subsp. *enterica* Inferred from Genome-Wide Reference-Free SNP Characters. doi: 10.1093/gbe/evt159 (2013).
6. Allard MW, Luo Y, Strain E, Li C, Keys CE, Son I, Stones R, Musser SM and BrownEW. High resolution clustering of *Salmonella enterica* serovar Montevideo strains using a next-generation sequencing approach. doi: 10.1186/1471-2164-13-32 (2013).
7. Ronholm J, Petronella N and Tamber S. *Salmonella enterica* Genome sequencing and assembly. PRJNA327743 (2016).
8. den Bakker HC, Moreno Switt AI, Govoni G, Cummings CA, Ranieri ML, Degoricija L, Hoelzer K, Rodriguez-Rivera LD, Brown S, Bolchacova E, Furtado MR and Wiedmann M. *Salmonella enterica* subsp. *enterica* serovar Montevideo str. S5-403. PRJNA59715 (2011).
9. Lienau EK, Strain E, Wang C, Zheng J, Ottesen AR, Keys CE, Hammack TS, Musser SM, Brown EW, Allard MW, Cao G, Meng J and Stones R. Identification of a salmonellosis outbreak by means of molecular sequencing. doi: 10.1056/NEJMc1100443 (2011).
10. Harhay DM, Bono JL, Smith TPL, Fields PI, Dinsmore BA, Santovinia M, Kelley CM, Wang R and Harhay GP Complete Closed Genome Sequences of *Salmonella enterica* subsp. *enterica* Serotypes Anatum, Montevideo, Typhimurium, and Newport, Isolated from Beef, Cattle, and Humans. doi: 10.1128/genomeA.01683-15 (2016).
11. Nguyen SV, Harhay DM, Bono JL, Smith TP, Fields PI, Dinsmore BA, Santovenia M, Wang R, Bosilevac JM and Harhay GP. Complete closed genome sequence of 11 *Salmonella enterica* subsp. *enterica* serovar Montevideo isolated from cattle and humans. CP017972 (2016). Data available on Figshare.
12. Nguyen SV, Harhay DM, Bono JL, Smith TP, Fields PI, Dinsmore BA, Santovenia M, Wang R, Bosilevac JM and Harhay GP. Complete closed genome sequence of 11 *Salmonella enterica* subsp. *enterica* serovar Montevideo isolated from cattle and humans. CP020752 (2016). Data available on Figshare.
13. Nguyen SV, Harhay DM, Bono JL, Smith TP, Fields PI, Dinsmore BA, Santovenia M, Wang R, Bosilevac JM and Harhay GP. Complete closed genome sequence of 11 *Salmonella enterica* subsp. *enterica* serovar Montevideo isolated from cattle and humans. CP017970 (2016). Data available on Figshare.
14. Nguyen SV, Harhay DM, Bono JL, Smith TP, Fields PI, Dinsmore BA, Santovenia M, Wang R, Bosilevac JM and Harhay GP. Complete closed genome sequence of 11 *Salmonella enterica* subsp. *enterica* serovar Montevideo isolated from cattle and humans. CP017973 (2016). Data available on Figshare.
15. Nguyen SV, Harhay DM, Bono JL, Smith TP, Fields PI, Dinsmore BA, Santovenia M, Wang R, Bosilevac JM and Harhay GP. Complete closed genome sequence of 11 *Salmonella enterica* subsp. *enterica* serovar Montevideo isolated from cattle and humans. CP017971 (2016). Data available on Figshare.
16. Nguyen SV, Harhay DM, Bono JL, Smith TP, Fields PI, Dinsmore BA, Santovenia M, Wang R, Bosilevac JM and Harhay GP. Complete closed genome sequence of 11 *Salmonella enterica* subsp. *enterica* serovar Montevideo isolated from cattle and humans. CP017978 (2016). Data available on Figshare.
17. Nguyen SV, Harhay DM, Bono JL, Smith TP, Fields PI, Dinsmore BA, Santovenia M, Wang R, Bosilevac JM and Harhay GP. Complete closed genome sequence of 11 *Salmonella enterica* subsp. *enterica* serovar Montevideo isolated from cattle and humans. CP017975 (2016). Data available on Figshare.
18. Nguyen SV, Harhay DM, Bono JL, Smith TP, Fields PI, Dinsmore, BA, Santovenia M, Wang R, Bosilevac JM and Harhay GP. Complete closed genome sequence of 11 *Salmonella enterica* subsp. *enterica* serovar Montevideo isolated from cattle and humans. CP017974 (2016). Data available on Figshare.
19. Nguyen SV, Harhay DM, Bono JL, Smith TP, Fields PI, Dinsmore BA, Santovenia M, Wang R, Bosilevac JM and Harhay GP Complete closed genome sequence of 11 *Salmonella enterica* subsp. *enterica* serovar Montevideo isolated from cattle and humans. CP017976 (2016). Data available on Figshare.
20. Nguyen SV, Harhay DM, Bono JL, Smith TP, Fields PI, Dinsmore BA, Santovenia M, Wang R, Bosilevac JM and Harhay GP. Complete closed genome sequence of 11 *Salmonella enterica* subsp. *enterica* serovar Montevideo isolated from cattle and humans. CP017977 (2016). Data available on Figshare.
21. Nguyen SV, Harhay DM, Bono JL, Smith TP, Fields PI, Dinsmore BA, Santovenia M, Wang R, Bosilevac JM and Harhay GP. Complete closed genome sequence of 11 *Salmonella enterica* subsp. *enterica* serovar Montevideo isolated from cattle and humans. CP020912 (2016). Data available on Figshare.

## Supplementary Data

Supplementary File 1
