# Supplementary material for: Comparative genomics of Salmonella enterica serovar Montevideo reveals lineage-specific gene differences that may influence ecological niche association
Source: Microb Genom. 2018 Jul 27;4(8):e000202. doi: 10.1099/mgen.0.000202 (PMC6159554; doi:10.1099/mgen.0.000202)
Supplement: Supplementary File 1 [file mgen-5-202-s001.pdf]

Fig. S1.

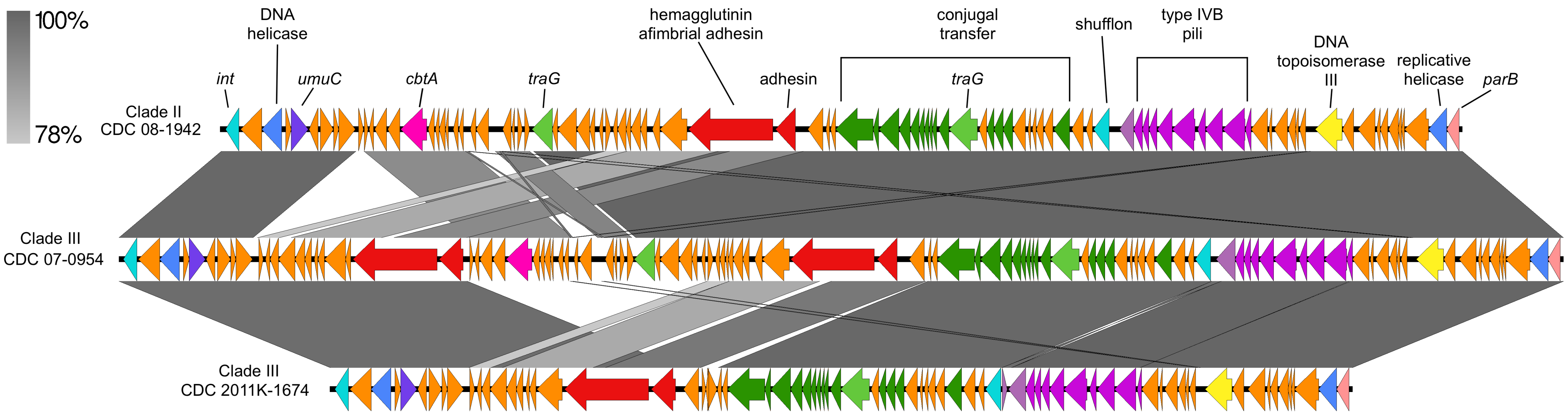

100%
78%
DNA
helicase
hemagglutinin
afimbrial adhesin
conjugal
transfer
shufflon
type IVB
pili
DNA
topoisomerase
III
replicative
helicase
int
umuC
cbtA
traG
adhesin
traG
parB
Clade II
CDC 08-1942
Clade III
CDC 07-0954
Clade III
CDC 2011K-1674

Table S1.

		Accession	Strain Name	Isolation source	Location	Year	Genome (bp)	Notes[a]
Clade I	1	AESY00000000	4441 H	Sunflower				
	2	MXWS01000000	BCW_2870					/collected_by="Technical University Of Denmark"
	3	LINX01000000	CVM N50432	Ground beef	USA: NM	2013		
	4	**CP017972**	USMARC-1904	Ground beef	BIFSCo Region 3	Jun-06	4598213	Pan-susceptible
	5	**CP020752**	CDC 2009K-0792	Human stool	USA: GA	4/11/09	4597370	Pan-susceptible
	6	**CP007540**	CDC 86-0391	Human clinical	USA:RI	Sep-86	4488371	Pan-susceptible
	7	LHGG01000000	CVM N45402	Ground beef	USA: MO	2013		
	8	**CP007222**	USMARC-1903	Ground beef	BIFSCo Region 6	Oct-06	4565462	Pan-susceptible
	9	MXCN01000000	CFSAN005876	Cattle		2006		
	10	**CP017970**	USMARC-1900	Bovine carcass hide	BIFSCo Region 3	Jun-05	4593026	Pan-susceptible
	11	LHKY01000000	CVM N48706	Ground beef	USA: LA	2013		
	12	**CP017973**	USMARC-1912	Bovine carcass hide	BIFSCo Region 3	Jul-05	4547966	Pan-susceptible
	13	JMMJ01000000	FVM_628064	Dog chew	USA: AR	13-Dec-06		
	14	LHKV01000000	CVM N48700	Ground beef	USA: LA	2013		
	15	**CP017971**	USMARC-1901	Bovine subiliac lymph node	BIFSCo Region 5	Aug-11	4587202	Pan-susceptible
	16	AOYQ01000000	ATCC 8387					
Clade II	17	AEST01000000	LQC 10					
	18	MATE01000000	SMo02					
	19	AESW01000000	29N	Soup				
	20	AFCS00000000	FSL S5-403					
	21	MXMR01000000	BCW_1602					/collected_by="FDA"
	22	AESU01000000	SARB30	Human clinical	USA: GA			
	23	MXXP01000000	BCW_2846					/collected_by="Technical University Of Denmark"
	24	MYBB01000000	BCW_2735	Human clinical	Taiwan	2002		/collected_by="Technical University Of Denmark"
	25	JYZL01000000	CVM N42336	Ground turkey	USA: GA	2012		
	26	JYUR01000000	CVM N30669	Ground turkey	USA: NM	2011		
	27	**CP017978**	CDC 2013K-0218	Human clinical	USA: VA	2013	4655223	Pan-susceptible
	28	**CP017975**	CDC 08-1942	Human clinical	USA: TX	9/11/08	4700953	Pan-susceptible
Clade III	29	AESS01000000	ATCC BAA710	Human clinical		1993		
	30	AESX01000000	42N	Human clinical				
	31	**CP017974**	CDC 07-0954	Human clinical	USA: NC	10/12/07	4773383	Pan-susceptible
	32	MXRG01000000	BCW_3982	Human clinical	USA: GA	2012		/collected_by="CDC"
	33	AESR01000000	SARB31	Human clinical	USA: FL			
	34	MXRJ01000000	BCW_3979	Human clinical	USA: MN	2011		
	35	**CP017976**	CDC 2011K-1674	Human clinical	USA: GA	9/15/11	4693862	Pan-susceptible
Clade IV	36	AETQ01000000	315731156	Pistachios				
	37	AHUX01000000	316111868	Pistachios		Mar-09		
	38	AESN01000000	531954	Pistachios				
	39	AESH00000000	315996572	Pistachios		2009		
	40	AESZ01000000	81038-01	Romaine lettuce				
	41	LHNX01000000	CVM N51288	Chicken breast	USA: NM	2013		
	42	AESQ00000000	CASC_09SCPH15965	Human clinical	USA: CA			
	43	MYET01000000	BCW_2615		USA			/collected_by="UC Davis CAHFS"
	44	MXQL01000000	BCW_4004	Human clinical	USA: CA	2012		/collected_by="CDC"
	45	AETD01000000	413180	Sea trout				
	46	AETE01000000	446600	King fish				
	47	AETB01000000	414877	Mozarella				
	48	AETC01000000	366867	Chinese perch				
	49	MATD01000000	SMo01					
	50	AESV01000000	19N	Chicken				
	51	**CP017977**	CDC 2012K-1544	Human clinical	USA: AR	10/16/12	4695308	Pan-susceptible
	52	AHHU01000000	CT_02035320	Pepper salami	USA			
	53	AHHV01000000	CT_02035321	Calabrese salami	USA			
	54	AHHS00000000	CT_02035278	Human clinical	USA			
	55	AHHT01000000	CT_02035318	Pepper salami	USA			
	56	**CP007530**	507440-20	Environmental swab	USA: RI	2/1/10	4694375	collected and sequenced by FDA
	57	AHHW01000000	CT_02035327	Food packaging	USA			
	58	AESI00000000	495297-1	Black pepper				
	59	AESK01000000	495297-4	Black pepper				
	60	AETO01000000	2009083312	Human clinical	USA: OH			
	61	AETH01000000	609460	Black pepper				
	62	AESL01000000	515920-1	Black pepper		2010		
	63	AETP01000000	2009085258	Human clinical	USA: OH			
	64	AESM01000000	515920-2	Black pepper		2010		
	65	AETJ01000000	556152	Red pepper				
	66	AESP01000000	OH_2009072675	Human clinical	USA: OH			
	67	AETW01000000	IA_2010008287	Lunch meat	USA: IA	2010		
	68	AETT01000000	IA_2010008283	Lunch meat	USA: IA	2010		
	69	AETS01000000	IA_2010008282	Food	USA: IA	22-Jan-10		
	70	AETR01000000	IA_2009159199	Human clinical	USA: IA			
	71	AETK01000000	MB101509-0077	Human clinical	USA: NC			
	72	AETF01000000	609458-1	Black pepper				
	73	AHHR01000000	80959-06	Iceburg lettuce head	USA	2010		
	74	AHIK01000000	IA_2010008286	Lunch meat	USA: IA	2010		
	75	AESJ01000000	495297-3	Black pepper				
	76	AETG01000000	556150-1	Red pepper				
	77	MAIW01000000	609458-2					
	78	**CP020912**	CDC 2010K-0257	Human clinical	USA: IL	2/12/10	4726140	contains plasmid pSMO-2010K-0257 (119867bp; CP020913); Pan-susceptible
	79	AETV01000000	IA_2010008285	Lunch meat	USA: IA	2010		
	80	AETA00000000	MD_MDA09249507	Human clinical	USA: MD			
	81	AETU01000000	IA_2010008284	Lunch meat	USA: IA			
	82	AETL01000000	MB102109-0047	Human clinical	USA: NC			
	83	AETM01000000	MB110209-0055	Human clinical	USA: NC			
	84	AESO01000000	NC_MB110209-0054	Human clinical	USA: NC	2009		
	85	AETN01000000	MB111609-0052	Human clinical	USA: NC			

[a] Antimicrobial susceptibility phenotypes where noted, were determined using the Sensititre broth microdilution method and CMV2AGNF plates which screen for susceptibility to 15 antimicrobial agents including: Amoxicillin/clavulanic acid; Ampicillin; Azithromycin; Cefoxitin; Ceftiofur; Ceftriaxone; Chloramphenicol; Ciprofloxacin; Gentamicin; Kanamycin; Nalidixic acid; Streptomycin; Sulfisoxazole;

Table S2. Mobile genetic elements of Montevideo. Prophages were identified using PHASTER and further visually inspected. Nucleotide locations for each phage within each genome are listed. PHASTER hits are shown with the 3 closest prophages and number of shared genes in parentheses. Phage regions are listed as SM Φ1-14 in order of appearance on the Montevideo pangenome.

Strain	Phage Region	Location	Size	CDS	G+C	PHASTER (Top 3 matches)
USMARC-1904	SM Φ5	1155595 - 1191128	35.5 kb	48	51.2	Fels-2 (39); ELPhiS (37); ENT90 (13)
	SM Φ10	3032984 - 3074508	41.5 kb	61	47.5	Salmon_118970_sal4 (22); g341c (14); ST160 (14)
CDC 2009K-0792	SM Φ5	1155680 - 1189925	34.2 kb	48	52.2	Fels-2 (39); ELPhiS (35); ENT90 (16)
	SM Φ10	3031794 - 3073527	41.7 kb	70	46.7	118970_sal4 (23); 118970_sal3 (15); vB_SemP_Emek (14)
CDC B94-007410	-	-	-	-	-	-
USMARC-1903	SM Φ7	2802508 - 2846360	48.9 kb	62	50.3	SfV (15); SEN34 (15); SfI (15)
USMARC-1900	SM Φ5	1192984 - 1227229	34.2 kb	48	52.2	Fels-2 (40); ELPhiS (36); ENT90 (16)
	SM Φ1	102124 - 139195	37.0 kb	53	51.7	D108 (45); SfMu (44); Mu (43)
USMARC-1912	SM Φ10	2983998 - 3024115	40.1 kb	60	46.9	103203_sal5 (19); SJ46 (15); g341c (13)
USMARC-1901	SM Φ2	679872 - 712288	32.4 kb	42	51.7	Coliphage 186 (38); PsP3 (32) SEN1 (28)
	SM Φ14	4370365 - 4402361	32.0 kb	44	51.6	P2 (32); Wphi (29); fiAA91_ss (29
CDC 08-1942	SM Φ5	1192984 - 1227229	35.4 kb	46	51.9	Fels-2 (40); ELPhiS (39); ENT90 (13)
	SM Φ3	679895 - 711355	31.5 kb	38	50.1	HP1 (18); phiO18P (17); HP2 (15)
CDC 2013K-0218	SM Φ5	1184843 - 1220219	35.4 kb	47	51.9	Fels-2 (39); ELPhiS (39); ENT90 (15)
	SM Φ3	679895 - 711355	31.5 kb	40	50.1	HP1 (18); phiO18P (17); HP2 (15)
	SM Φ6	679872 - 712288	46.9 kb	75	48.7	vB_SosS_Oslo (28); SPN3UB (22); ES18 (19)
CDC 07-0954	SM Φ8	2894155 - 2937783	43.6 kb	62	50.0	SEN34 (30); Gifsy-2 (17); Gifsy-1 (14)
	SM Φ12	3204153 - 3248023	43.9 kb	54	52.0	Gifsy-2 (19); mEp460 (14); cdtI (14)
	SM Φ13	3475575 - 3515923	40.3 kb	54	54.9	D108 (30); Mu (30); SfMu (29)
CDC 2011K-1674	SM Φ9	2900652 - 2951713	51.1 kb	65	49.9	Gifsy-1 (34); Gifsy-2 (26); SPN3UB (13)
	SM Φ4	1049487 - 1087571	38.1 kb	55	55.1	D108 (32); SfMu (32); Mu (31)
CDC 2012K-1544	SM Φ5	1184874 - 1220243	35.3 kb	46	51.9	Fels-2 (41); Elphis (38); ENT90 (13)
	SM Φ6	1515653 - 1562597	48.0 kb	70	48.7	vB_SosS_Oslo (28); ES18 (24); SPN3UB (21)
	SM Φ11	3152760 - 3185106	32.3 kb	38	38.4	EcoS_NBD2 (3); HK639 (3); vB_CsaM_GAP32 (2)
CDC 2010K-0257	SM Φ5	1182074 - 1217412	35.3 kb	48	51.9	Fels-2 (41); ELPhiS (38); ENT90 (13)
	SM Φ6	1513293 - 1561278	48.0 kb	76	48.7	vB_SosS_Oslo (26); ES18 (24); SPN3UB (21)

Table S3. Metabolic island differences among the Montevideo clades as well as locus tag IDs for genes delineating each metabolic island.

Strain	®-glucuronidase (*gus*)	Aldo/Keto Sugar Kinase (*ydj*)	PTS Tagatose (*tag*)	L-Rhamnonate Metabolism (*yfa*)	Fructose-like PTS (*frw*)	Allantoin Metabolism (*all*)	Inositol Metabolism (*ino*)	Quinate/shikimate utilization (*ydi*)
S. Typhimurium LT2	null	null	STM3252-STM3256	STM2289-STM2292	STM4110-STM4116	STM0514-STM0532	STM4417-STM4436	STM1350-STM1362
USMARC-1904	AW68_03800-AW68_03815	AW68_13185-AW68_13220	AW68_03120-AW68_03145	AW68_07895-AW68_07910				
CDC 2009K-0792	AW73_03800-AW73_03815	AW73_13175-AW73_13210	AW73_03120-AW73_03145	AW73_07890-AW73_07905				
CDC 86-0391	AW70_7340-AW70_7370	AW70_24280-AW70_24350	AW70_6060-AW70_6110	AW70_14770-AW70_14800				
USMARC-1903	AW67_36410-AW67_36440	AW67_19200-AW67_19130	AW67_37730-AW67_37680	AW67_29040-AW67_29010				
USMARC-1900	AW63_04065-AW63_04080	AW63_13445-AW63_13480	AW63_03385-AW63_03415	AW63_08160-AW63_08175				
USMARC-1912	AW64_03815-AW64_03830	AW64_12925-AW64_12960	AW64_03135-AW64_03165	AW64_07685-AW64_07700				
USMARC-1901	AW47_04010-AW47_04025	AW47_13145-AW47_13180	AW47_03120-AW47_03150	AW47_07860-AW47_07875				
CDC 08-1942	AW72_03825-AW72_03840	AW72_12685-AW72_12720	AW72_03010-AW72_03040	AW72_07725-AW72_07740	AW72_21480-AW72_21505	AW72_16065-AW72_16150		
CDC 2013K-0218	AW76_04000-AW76_04015	AW76_13760-AW76_13795	AW76_03120-AW76_03145	AW76_08470-AW76_08485	AW76_22430-AW76_22455	AW76_17265-AW76_17350		
CDC 07-0954	AW71_03615-AW71_03630					AW71_16700-AW71_16785		
CDC 2011K-1674	AW74_03780-AW74_03795					AW74_17360-AW74_17445		
CDC 2012K-1544	AW77_03910-AW73_03925						AW77_20925-AW77_21020	AW77_13510-AW77_13570
CDC 2010K-0257	AW75_03750-AW75_03765						AW75_20100-AW75_20190	AW75_12805-AW75_12865

Table S4. Locus tag IDs for all secreted effectors in the Montevideo strains sequenced identified by BLAST analysis using the virulence finder database (VFDB), as well as function if known.

LT2 reference unless noted otherwise in parentheses	USMARC-1904	CDC 2009K-0792	CDC 86-0391	USMARC-1903	USMARC-1900	USMARC-1912	USMARC-1901	CDC 08-1942	CDC 2013K-0218	CDC 07-0954	CDC 2011K-1674	CDC 2010K-0257	CDC 2012K-1544	T3SS1/2	Function	Ref
avrA/yopJ	-	-	-	-	-	-	-	-	-	-	-	AW75_05135	AW77_05375	1	anti-inflammatory, inhibit NF-kb	(1)
orgC	AW68_14145	AW73_05240	AW70_10210	AW67_33590	AW63_05505	AW64_05255	AW47_05450	AW72_05195	AW76_05445	AW71_05530	AW74_05890	AW75_05120	AW77_05355	1	unknown	(2)
sipA	AW68_05170	AW73_05170	AW70_10070	AW67_33730	AW63_05435	AW64_05185	AW47_05380	AW72_05125	AW76_05375	AW71_05460	AW74_05820	AW75_05050	AW77_05285	1	actin polymerization, bacterial entry	(2-4)
sipB	AW68_05155	AW73_05155	AW70_10040	AW67_33760	AW63_05420	AW64_05170	AW47_05365	AW72_05110	AW76_05360	AW71_05445	AW74_05805	AW75_05035	AW77_05270	1	T3SS translocon	(2-4)
sipC	AW68_05160	AW73_05160	AW70_10050	AW67_33750	AW63_05425	AW64_05175	AW47_05370	AW72_05115	AW76_05365	AW71_05450	AW74_05810	AW75_05040	AW77_05275	1	F-actin nucleation	(2-4)
sipD	AW68_05165	AW73_05165	AW70_10060	AW67_33740	AW63_05430	AW64_05180	AW47_05375	AW72_05120	AW76_05370	AW71_05455	AW74_05815	AW75_05045	AW77_05280	1	T3SS regulation	(5)
sopA	AW68_08990	AW73_08980	AW70_16800	AW67_27040	AW63_09250	AW64_08730	AW47_08950	AW72_08735	AW76_09570	AW71_08820	AW74_09370	AW75_08995	AW77_09460	1	ubiquitin ligase	(3, 4)
sopB	AW68_14425	AW73_14415	AW70_26580	AW67_16900	AW63_14685	AW64_14165	AW47_14385	AW72_13855	AW76_15000	AW71_13890	AW74_14765	AW75_14275	AW77_15070	1	inositol phosphate phosphatase	(2-4)
sopE (SL1344)	-	-	-	AW67_16530	-	-	-	-	-	-	-	-	-	1	activate Rho GTPases	(6, 7)
sopE2	AW68_10170	AW73_10160	AW70_18970	AW67_24870	AW63_10430	AW64_09910	AW47_10130	AW72_09840	AW76_10750	AW71_09925	AW74_10550	AW75_10095	AW77_10640	1	activate Rho GTPases	(6, 7)
sptP	AW68_05190	AW73_05190	AW70_10110	AW67_33690	AW63_05455	AW64_05205	AW47_05400	AW72_05145	AW76_05395	AW71_05480	AW74_05840	AW75_05070	AW77_05305	1	anti-inflammatory; reverse actin reorgnanization	(2-4)
slrP	AW68_15420	AW73_15410	AW70_28520	AW67_14340	AW63_15680	AW64_15160	AW47_15380	AW72_14835	AW76_16010	AW71_15195	AW74_16100	AW75_15240	AW77_16065	1,2	ubiquitin ligase, apoptosis	(2-4)
sopD	AW68_04840	AW73_04840	AW70_9350	AW67_34440	AW63_05105	AW64_04855	AW47_05050	AW72_04795	AW76_05040	AW71_05130	AW74_05215	AW75_04720	AW77_04950	1,2	fluid secretion, macropinocytosis	(2-4)
steA	AW68_11700	AW73_11690	AW70_21750	AW67_22070	AW63_11960	AW64_11440	AW47_11660	AW72_11275	AW76_12280	AW71_11355	AW74_12085	AW75_11530	AW77_12165	1,2	long-term macrophage intracellular survival	(8)
gogB	-	-	-	-	-	-	-	-	-	-	-	-	-	2	anti-inflammatory	(4)
pipA	AW68_14445	AW73_14435	AW70_26620	AW67_16860	AW63_14705	AW64_14185	AW47_14405	AW72_13880	AW76_15020	AW71_13915	AW74_14785	AW75_14300	AW77_15090		Target NF-κB, anti-inflammatory	(9, 10)
pipB	AW68_14440	AW73_14430	AW70_26610	AW67_16870	AW63_14700	AW64_14180	AW47_14400	AW72_13875	AW76_15015	AW71_13910	AW74_14780	AW75_14295	AW77_15085	2	Salmonella-induced filament (Sif) induction	(11, 12)
pipB2	AW68_05740	AW73_05740	AW70_11110	AW67_32700	AW63_06005	AW64_05755	AW47_05950	AW72_05660	AW76_05940	AW71_06000	AW74_06385	AW75_05585	AW77_05855	2	Sif extension	(11)
sifA	AW68_13685	AW73_13675	AW70_25200	AW67_18290	AW63_13945	AW64_13425	AW47_13645	AW72_13155	AW76_14260	AW71_13190	AW74_14030	AW75_13570	AW77_14335	2	Sif, Salmonella containing vacuolose (SCV) formation	(3, 4)
sifB	AW68_11600	AW73_11590	AW70_21570	AW67_22250	AW63_11860	AW64_11340	AW47_13645	AW72_11175	AW76_12185	AW71_11255	AW74_11985	AW75_11430	AW77_12065	2	Sif formation	(4, 12)
sopD2	AW68_14750	AW73_14740	AW70_27230	AW67_15630	AW63_15010	AW64_14490	AW47_14710	AW72_14185	AW76_15340	AW71_14525	AW74_15415	AW75_14600	AW77_15395	2	Sif formation	(13)

spiC/ssaB	AW68_12725	AW73_12715	AW70_44225	AW67_20080	AW63_12985	AW64_12465	AW47_12685	AW72_12255	AW76_13300	AW71_12335	AW74_13110	AW75_12655	AW77_13355	2	regulates Sif formation	(3, 4)
spv	-	-	-	-	-	-	-	-	-	-	-	-	-	2	actin depolymerization, inactivate MAPK	(3, 4, 14)
sseB	AW68_12700	AW73_12690	AW70_23370	AW67_20110	AW63_12960	AW64_12440	AW47_12660	AW72_12230	AW76_13275	AW71_12310	AW74_13085	AW75_12630	AW77_13325	2	pore formation	(3, 4)
sseC	AW68_12690	AW73_12680	AW70_23350	AW67_20130	AW63_12950	AW64_12430	AW47_12650	AW72_12220	AW76_13265	AW71_12300	AW74_13075	AW75_12620	AW77_13315	2	pore formation	(3, 4)
sseD	AW68_12685	AW73_12675	AW70_23340	AW67_20140	AW63_12945	AW64_12425	AW47_12645	AW72_12215	AW76_13260	AW71_12295	AW74_13070	AW75_12615	AW77_13310	2	pore formation	(3, 4)
sseE	AW68_12680	AW73_12670	AW70_23330	AW67_20150	AW63_12940	AW64_12420	AW47_12640	AW72_12210	AW76_13255	AW71_12290	AW74_13065	AW75_12610	AW77_13305	2	unknown	(15)
sseF	AW68_12670	AW73_12660	AW70_23310	AW67_20170	AW63_12930	AW64_12410	AW47_12630	AW72_12200	AW76_13245	AW71_12280	AW74_13055	AW75_12600	AW77_13295	2	SCV positioning	(3, 4)
sseG	AW68_12665	AW73_12655	AW70_23300	AW67_20180	AW63_12925	AW64_12405	AW47_12625	AW72_12195	AW76_13240	AW71_12275	AW74_13050	AW75_12595	AW77_13290	2	Sif formation	(3, 4)
sseI	-	-	-	-	-	-	-	-	-	-	-	-	-	2	migration of actin filaments around SCV	(3, 4, 12)
sseJ	AW68_11450	AW73_11440	AW70_21290	AW67_22540	AW63_11710	AW64_11190	AW47_11410	AW72_11035	AW76_12035	AW71_12275	AW74_11835	AW75_11290	AW77_11915	2	SCV positioning, targeting to perinuclear	(3, 4)
sseK1	AW68_21865	AW73_21915	AW70_40290	AW67_2520	AW63_21830	AW64_21605	AW47_21565	AW72_21285	AW76_22210	AW71_21765	AW74_22335	AW75_21450	AW77_22320	2	Sif formation	(4, 12)
sseK2/NleB	-	-	-	-	-	-	-	-	-	-	-	-	-	2	Sif formation	(4, 12)
sseL	AW68_07920	AW73_07915	AW70_14820	AW67_28990	AW63_08185	AW64_07710	AW47_07885	AW72_07745	AW76_08495	AW71_07835	AW74_08310	AW75_08010	AW77_08400	2	deubiquinates, anti-inflammatory	(4)
sspH1 (14028S)	-	-	-	-	-	-	-	-	-	-	-	-	-	2	inhibits NF-kB, downregulates IL-8	(4)
sspH2	-	-	-	-	-	-	-	-	-	-	-	-	-	2	E3 ubiquitin ligase	(4)
steB	AW68_11460	AW73_11450	AW70_44105	2349729-2349328	AW63_11720	AW64_11200	AW47_11420	2283045-2283446	AW76_12045	2315437-2315838	AW74_11845	2323345-2323746	AW77_11925	2	unknown	(2, 4)
steC	AW68_11040	AW73_11030	AW70_20550	AW67_23290	AW63_11300	AW64_10780	AW47_11000	AW72_10655	AW76_11625	AW71_10740	AW74_11425	AW75_10910	AW77_11505	2	kinase with activity on actin network	(4)

Table S5. Locus tag IDs of Montevideo virulence genes identified by BLAST analysis using the virulence finder database (VFDB), as well as function if known.

**LT2 reference unless noted otherwise in parentheses**	USMARC-1904	CDC 2009K-0792	CDC 86-0391	USMARC-1903	USMARC-1900	USMARC-1912	USMARC-1901	CDC 08-1942	CDC 2013K-0218	CDC 07-0954	CDC 2011K-1674	CDC 2010K-0257	CDC 2012K-1544	Function	Ref.
cdtB (CT18)	AW68_13520	AW73_13510	AW70_24900	AW67_18590	AW63_13780	AW64_13260	AW47_13480	AW72_13015	AW76_14095	AW71_13050	AW74_13865	AW75_13425	AW77_14170	cell cycle arrest	(16, 17)
clyA/hlyE (CT18)	AW68_11815	AW73_11805	AW70_21980	AW67_21840	AW63_12075	AW64_11555	AW47_11775	AW72_11385	AW76_12395	AW71_11465	AW74_12200	AW75_11640	AW77_12280	pore formation	(18)
gtgE	-	-	-	-	-	-	-	-	-	AW71_14070	AW74_14940	-	-	prevent Rab29 recruitment, protease	(2, 4, 19)
msgA	AW68_13510	AW73_13500	AW70_24880	AW67_18610	AW63_13770	AW64_13250	AW47_13470	AW72_13005	AW76_14085	AW71_13040	AW74_13855	AW75_13415	AW77_14160	unknown	(20)
pltA/artA (CT18)	AW68_13535	AW73_13525	AW70_24930	AW67_18560	AW63_13795	AW64_13275	AW47_13495	AW72_13030	AW76_14110	AW71_13065	AW74_13880	AW75_13440	AW77_14185	ADP-ribosylation of host protein	(16, 17)
pltB/artB (CT18)	AW68_13540	AW73_13530	AW70_44340	2607280-2607693	AW63_13800	AW64_13280	AW47_13500	AW72_13035	AW76_14115	AW71_13070	AW74_13885	AW75_13445	AW77_14190	ADP-ribosylation of host protein	(16, 17)
sciR	AW68_18155	AW73_18200	AW70_33140 / AW70_33150	AW67_9700 / AW67_9710	AW63_18120	AW64_17895	AW47_17835	AW72_17280	AW76_18540	AW71_18210	AW74_18635	AW75_17810	AW77_18525	putative shiga-like toxin	(21)
sodCI	-	-	-	-	-	-	-	-	-	-	-	-	-	Protection from phagocyte oxidative burst	(22)

1. **Wu H, Jones RM, Neish AS.** 2012. The Salmonella effector AvrA mediates bacterial intracellular survival during infection in vivo. Cell Microbiol **14:**28-39.
2. **Elhadad D, Desai P, Grassl GA, McClelland M, Rahav G, Gal-Mor O.** 2016. Differences in Host Cell Invasion and Salmonella Pathogenicity Island 1 Expression between Salmonella enterica Serovar Paratyphi A and Nontyphoidal S. Typhimurium. Infect Immun **84:**1150-1165.
3. **Srikanth CV, Mercado-Lubo R, Hallstrom K, McCormick BA.** 2011. Salmonella effector proteins and host-cell responses. Cell Mol Life Sci **68:**3687-3697.
4. **Ramos-Morales F.** 2012. Impact of Salmonella enterica Type III Secretion System Effectors on the Eukaryotic Host Cell. ISRN Cell Biology **2012:**36.
5. **Glasgow AA, Wong HT, Tullman-Ercek D.** 2017. A Secretion-Amplification Role for Salmonella enterica Translocon Protein SipD. ACS Synth Biol **6:**1006-1015.
6. **Zhang S, Santos RL, Tsolis RM, Mirold S, Hardt WD, Adams LG, Baumler AJ.** 2002. Phage mediated horizontal transfer of the sopE1 gene increases enteropathogenicity of Salmonella enterica serotype Typhimurium for calves. FEMS Microbiol Lett **217:**243-247.

7. **Pelludat C, Mirold S, Hardt WD.** 2003. The SopEPhi phage integrates into the ssrA gene of Salmonella enterica serovar Typhimurium A36 and is closely related to the Fels-2 prophage. J Bacteriol **185:**5182-5191.
8. **McQuate SE, Young AM, Silva-Herzog E, Bunker E, Hernandez M, de Chaumont F, Liu X, Detweiler CS, Palmer AE.** 2017. Long-term live-cell imaging reveals new roles for Salmonella effector proteins SseG and SteA. Cell Microbiol **19**.
9. **Wood MW, Jones MA, Watson PR, Hedges S, Wallis TS, Galyov EE.** 1998. Identification of a pathogenicity island required for Salmonella enteropathogenicity. Mol Microbiol **29:**883-891.
10. **Sun H, Kamanova J, Lara-Tejero M, Galan JE.** 2016. A Family of Salmonella Type III Secretion Effector Proteins Selectively Targets the NF-kappaB Signaling Pathway to Preserve Host Homeostasis. PLoS Pathog **12:**e1005484.
11. **Knodler LA, Vallance BA, Hensel M, Jackel D, Finlay BB, Steele-Mortimer O.** 2003. Salmonella type III effectors PipB and PipB2 are targeted to detergent-resistant microdomains on internal host cell membranes. Mol Microbiol **49:**685-704.
12. **Rajashekar R, Liebl D, Chikkaballi D, Liss V, Hensel M.** 2014. Live cell imaging reveals novel functions of Salmonella enterica SPI2-T3SS effector proteins in remodeling of the host cell endosomal system. PLoS One **9:**e115423.
13. **D'Costa VM, Braun V, Landekic M, Shi R, Proteau A, McDonald L, Cygler M, Grinstein S, Brumell JH.** 2015. Salmonella Disrupts Host Endocytic Trafficking by SopD2-Mediated Inhibition of Rab7. Cell Rep **12:**1508-1518.
14. **Rotger R, Casadesus J.** 1999. The virulence plasmids of Salmonella. Int Microbiol **2:**177-184.
15. **Hensel M, Shea JE, Waterman SR, Mundy R, Nikolaus T, Banks G, Vazquez-Torres A, Gleeson C, Fang FC, Holden DW.** 1998. Genes encoding putative effector proteins of the type III secretion system of Salmonella pathogenicity island 2 are required for bacterial virulence and proliferation in macrophages. Mol Microbiol **30:**163-174.
16. **Miller RA, Wiedmann M.** 2016. The Cytolethal Distending Toxin Produced by Nontyphoidal Salmonella Serotypes Javiana, Montevideo, Oranienburg, and Mississippi Induces DNA Damage in a Manner Similar to That of Serotype Typhi. MBio **7**.
17. **Rodriguez-Rivera LD, Bowen BM, den Bakker HC, Duhamel GE, Wiedmann M.** 2015. Characterization of the cytolethal distending toxin (typhoid toxin) in non-typhoidal Salmonella serovars. Gut Pathog **7:**19.
18. **von Rhein C, Bauer S, Lopez Sanjurjo EJ, Benz R, Goebel W, Ludwig A.** 2009. ClyA cytolysin from Salmonella: distribution within the genus, regulation of expression by SlyA, and pore-forming characteristics. Int J Med Microbiol **299:**21-35.
19. **Ho TD, Figueroa-Bossi N, Wang M, Uzzau S, Bossi L, Slauch JM.** 2002. Identification of GtgE, a novel virulence factor encoded on the Gifsy-2 bacteriophage of Salmonella enterica serovar Typhimurium. J Bacteriol **184:**5234-5239.
20. **Gunn JS, Alpuche-Aranda CM, Loomis WP, Belden WJ, Miller SI.** 1995. Characterization of the Salmonella typhimurium pagC/pagD chromosomal region. J Bacteriol **177:**5040-5047.
21. **Mohammed M, Cormican M.** 2016. Whole genome sequencing provides insights into the genetic determinants of invasiveness in Salmonella Dublin. Epidemiol Infect **144:**2430-2439.
22. **Sly LM, Guiney DG, Reiner NE.** 2002. Salmonella enterica serovar Typhimurium periplasmic superoxide dismutases SodCI and SodCII are required for protection against the phagocyte oxidative burst. Infect Immun **70:**5312-5315.

Table S6. Locus tag IDs (if annotated, otherwise genome coordinates are indicated) of genes within *fim* operons identified in Montevideo sequences.

		**USMARC-1904**	CDC 2009K-0792	CDC 86-0391	USMARC-1903	USMARC-1900	USMARC-1912	USMARC-1901	**CDC 08-1942**	CDC 2013K-0218	**CDC 07-0954**	CDC 2011K-1674	**CDC 2012K-1544**	CDC 2010K-0257
sdg	*A*	AW68_18690	AW73_18735	AW70_34100	AW67_8740	AW63_18655	AW64_18430	AW47_18370	AW72_17785	AW76_19075	AW71_18710	AW74_19150	AW75_18310	AW77_19040
	B	AW68_18695	AW73_18740	AW70_34110	AW67_8730	AW63_18660	AW64_18435	AW47_18375	AW72_17790	AW76_19080	AW71_18715	AW74_19155	AW75_18315	AW77_19045
	C	AW68_18700	AW73_18745	AW70_34120	AW67_8720	AW63_18665	AW64_18440	AW47_18380	AW72_17795	AW76_19085	AW71_18720	AW74_19160	AW75_18320	AW77_19050
	D	AW68_18705	AW73_18750	AW70_34130	AW67_8710	AW63_18670	AW64_18445	AW47_18385	AW72_17800	AW76_19090	AW71_18725	AW74_19165	AW75_18325	AW77_19055
	E	AW68_18710	AW73_18755	AW70_34140	AW67_8700	AW63_18675	AW64_18450	AW47_18390	AW72_17805	AW76_19095	AW71_18730	AW74_19170	AW75_18330	AW77_19060
	F	AW68_18715	AW73_18760	AW70_34150	AW67_8690	AW63_18680	AW64_18455	AW47_18395	AW72_17810	AW76_19100	AW71_18735	AW74_19175	AW75_18335	AW77_19065
	G	AW68_18720	AW73_18765	AW70_34160	AW67_8680	AW63_18685	AW64_18460	AW47_18400	AW72_17815	AW76_19105	AW71_18740	AW74_19180	AW75_18340	AW77_19070
fae	*A*	AW68_21080	AW73_21130	AW70_38780	AW67_4040	AW63_21045	AW64_20820	AW47_20780	AW72_20520	AW76_21420	AW71_20995	AW74_21545	AW75_20680	AW77_21525
	B	AW68_21130	AW73_21180	AW70_38870	AW67_3940	AW63_21095	AW64_20870	AW47_20830	AW72_20565	AW76_21470	AW71_21040	AW74_21595	AW75_20725	AW77_21575
	C	AW68_21125	AW73_21175	AW70_38860	AW67_3950	AW63_21090	AW64_20865	AW47_20825	AW72_20560	AW76_21465	AW71_21035	AW74_21590	AW75_20720	AW77_21570
	D	AW68_21120	AW73_21170	AW70_38850	AW67_3960	AW63_21085	AW64_20860	AW47_20820	AW72_20555	AW76_21460	AW71_21030	AW74_21585	AW75_20715	AW77_21565
	E	AW68_21115	AW73_21165	AW70_38840	AW67_3970	AW63_21080	AW64_20855	AW47_20815	AW72_20550	AW76_21455	AW71_21025	AW74_21580	AW75_20710	AW77_21560
	F	AW68_21110	AW73_21160	AW70_38830	AW67_3980	AW63_21075	AW64_20850	AW47_20810	AW72_20545	AW76_21450	AW71_21020	AW74_21575	AW75_20705	AW77_21555
	G	AW68_21105	AW73_21155	AW70_38820	AW67_3990	AW63_21070	AW64_20845	AW47_20805	AW72_20540	AW76_21445	AW71_21015	AW74_21570	AW75_20700	AW77_21550
	H	AW68_21100	AW73_21150	AW70_38810	AW67_4000	AW63_21065	AW64_20840	AW47_20800	AW72_20535	AW76_21440	AW71_21010	AW74_21565	AW75_20695	AW77_21545
	J	AW68_21095	AW73_21145	AW70_38800	4105860-4105096	AW63_21060	AW64_20835	AW47_20795	AW72_20530	AW76_21435	AW71_21005	AW74_21560	AW75_20690	AW77_21540
	K	AW68_21090	AW73_21140	AW70_45215	4105109-4104675	AW63_21055	AW64_20830	AW47_20790	4235682-4235248	AW76_21430	4314393-4313960	AW74_21555	4268500-4268066	AW77_21535
sdf	*A*	AW68_13820	AW73_13810	AW70_25480	AW67_18000	AW63_14080	AW64_13560	AW47_13780	AW72_13290	AW76_14395	AW71_13325	AW74_14165	AW75_13705	AW77_14470
	B	AW68_13825	AW73_13815	AW70_25490	AW67_17990	AW63_14085	AW64_13565	AW47_13785	AW72_13295	AW76_14400	AW71_13330	AW74_14170	AW75_13710	AW77_14475
	C	AW68_13830	AW73_13820	AW70_25500	AW67_17980	AW63_14090	AW64_13570	AW47_13790	AW72_13300	AW76_14405	AW71_13335	AW74_14175	AW75_13715	AW77_14480
	D	AW68_13835	AW73_13825	AW70_25510	AW67_17970	AW63_14095	AW64_13575	AW47_13795	AW72_13305	AW76_14410	AW71_13340	AW74_14180	AW75_13720	AW77_14485
	E	AW68_13840	AW73_13830	AW70_25520	AW67_17960	AW63_14100	AW64_13580	AW47_13800	AW72_13310	AW76_14415	AW71_13345	AW74_14185	AW75_13725	AW77_14490
	F	AW68_13845	AW73_13835	AW70_25530	AW67_17950	AW63_14105	AW64_13585	AW47_13805	AW72_13315	AW76_14420	AW71_13350	AW74_14190	AW75_13730	AW77_14495
peh	*A*	AW68_08590	AW73_08580	AW70_43680	1691388-1691930	AW63_08850	AW64_08375	AW47_08550	AW72_08350	AW76_09155	AW71_08440	AW74_08970	AW75_08615	AW77_09060
	B	AW68_08595	AW73_08585	AW70_16020	AW67_27810	AW63_08855	AW64_08380	AW47_08555	AW72_08355	AW76_09160	AW71_08445	AW74_08975	AW75_08620	AW77_09065
	C	AW68_08600	AW73_08590	AW70_16030	AW67_27800	AW63_08860	AW64_08385	AW47_08560	AW72_08360	AW76_09165	AW71_08450	AW74_08980	AW75_08625	AW77_09070
	D	AW68_08605	AW73_08595	AW70_16040	AW67_27790	AW63_08865	AW64_08390	AW47_08565	AW72_08365	AW76_09170	AW71_08455	AW74_08985	AW75_08630	AW77_09075
	E	AW68_08610	AW73_08600	AW70_16050	AW67_27780	AW63_08870	AW64_08395	AW47_08570	AW72_08370	AW76_09175	AW71_08460	AW74_08990	AW75_08635	AW77_09080
sti	*A*	AW68_18755	AW73_18805	AW70_34230	AW67_8610	AW63_18720	AW64_18495	AW47_18435	AW72_17845	AW76_19145	AW71_18770	AW74_19215	AW75_18370	AW77_19110
	B	AW68_18760	AW73_18810	AW70_34240	AW67_8600	AW63_18725	AW64_18500	AW47_18440	AW72_17850	AW76_19150	AW71_18775	AW74_19220	AW75_18375	AW77_19115
	C	AW68_18765	AW73_18815	AW70_34250	AW67_8590	AW63_18730	AW64_18505	AW47_18445	AW72_17855	AW76_19155	AW71_18780	AW74_19225	AW75_18380	AW77_19120
	H	AW68_18770	AW73_18820	AW70_34260	AW67_8580	AW63_18735	AW64_18510	AW47_18450	AW72_17860	AW76_19160	AW71_18785	AW74_19230	AW75_18385	AW77_19125
stb	*A*	AW68_17875	AW73_17920	AW70_32610	AW67_10230	AW63_17840	AW64_17615	AW47_17555	AW72_17015	AW76_18255	AW71_17915	AW74_18340	AW75_17520	AW77_18210
	B	AW68_17880	AW73_17925	AW70_32620	AW67_10240	AW63_17845	AW64_17620	AW47_17560	AW72_17020	AW76_18260	AW71_17920	AW74_18345	AW75_17525	AW77_18215
	C	AW68_17885	AW73_17930	AW70_32630	AW67_10220	AW63_17850	AW64_17625	AW47_17565	AW72_17025	AW76_18265	AW71_17925	AW74_18350	AW75_17530	AW77_18220
	D	AW68_17890	AW73_17935	AW70_32640	AW67_10210	AW63_17855	AW64_17630	AW47_17570	AW72_17030	AW76_18270	AW71_17930	AW74_18355	AW75_17535	AW77_18225
	E	AW68_17895	AW73_17940	AW70_32650	AW67_10200	AW63_17860	AW64_17635	AW47_17575	AW72_17035	AW76_18275	AW71_17935	AW74_18360	AW75_17540	AW77_18230
saf	*A*	AW68_18090	AW73_18135	AW70_44850	3491673-3492179	AW63_18055	AW64_17830	AW47_17770	AW72_17220	AW76_18475	AW71_18110	AW74_18555	AW75_17720	AW77_18430
	B	AW68_18085	AW73_18130	AW70_33000	AW67_9840	AW63_18050	AW64_17825	AW47_17765	AW72_17215	AW76_18470	AW71_18105	AW74_18550	AW75_17715	AW77_18425
	C	AW68_18080	AW73_18125	AW70_32990	AW67_9850	AW63_18045	AW64_17820	AW47_17760	AW72_17210	AW76_18465	AW71_18100	AW74_18545	AW75_17710	AW77_18420
	D	AW68_18075	AW73_18120	AW70_32980	AW67_9860	AW63_18040	AW64_17815	AW47_17755	AW72_17205	AW76_18460	AW71_18095	AW74_18540	AW75_17705	AW77_18415
bcf	*A*	AW68_19575	AW73_19625	AW70_35860	AW67_6970	AW63_19540	AW64_19315	AW47_19275	AW72_18630	AW76_19970	AW71_19550	AW74_20035	AW75_19145	AW77_19925
	B	AW68_19570	AW73_19620	AW70_35850	AW67_6980	AW63_19535	AW64_19310	AW47_19270	AW72_18625	AW76_19965	AW71_19545	AW74_20030	AW75_19140	AW77_19920
	C	AW68_19565	AW73_19615	AW70_35840	AW67_6990	AW63_19530	AW64_19305	AW47_19265	AW72_18620	AW76_19960	AW71_19540	AW74_20025	AW75_19135	AW77_19915
	D	AW68_19560	AW73_19610	AW70_35830	AW67_7000	AW63_19525	AW64_19300	AW47_19260	AW72_18615	AW76_19955	AW71_19535	AW74_20020	AW75_19130	AW77_19910
	E	AW68_19555	AW73_19605	AW70_35820	AW67_7010	AW63_19520	AW64_19295	AW47_19255	AW72_18610	AW76_19950	AW71_19530	AW74_20015	AW75_19125	AW77_19905
	F	AW68_19550	AW73_19600	AW70_35810	AW67_7020	AW63_19515	AW64_19290	AW47_19250	AW72_18605	AW76_19945	AW71_19525	AW74_20010	AW75_19120	AW77_19900
	G	AW68_19545	AW73_19595	AW70_35800	AW67_7030	AW63_19510	AW64_19285	AW47_19245	AW72_18600	AW76_19940	AW71_19520	AW74_20005	AW75_19115	AW77_19895
fim	*A*	AW68_16905	AW73_16945	AW70_30710	AW67_12140	AW63_16865	AW64_16645	AW47_16580	AW72_16010	AW76_17200	AW71_16645	AW74_17295	AW75_16605	AW77_17255
	C	AW68_16895	AW73_16935	AW70_30690	AW67_12160	AW63_16855	AW64_16635	AW47_16570	AW72_16000	AW76_17190	AW71_16635	AW74_17285	AW75_16595	AW77_17245
	D	AW68_16890	AW73_16930	AW70_30680	AW67_12170	AW63_16850	AW64_16630	AW47_16565	AW72_15995	AW76_17185	AW71_16630	AW74_17280	AW75_16590	AW77_17240
	F	AW68_16880	AW73_16920	AW70_30660	AW67_12190	AW63_16840	AW64_16620	AW47_16555	AW72_15985	AW76_17175	AW71_16620	AW74_17270	AW75_16580	AW77_17230
	H	AW68_16885	AW73_16925	AW70_30670	AW67_12180	AW63_16845	AW64_16625	AW47_16560	AW72_15990	AW76_17180	AW71_16625	AW74_17275	AW75_16585	AW77_17235
	I	AW68_16900	AW73_16940	AW70_30700	AW67_12150	AW63_16860	AW64_16640	AW47_16575	AW72_16005	AW76_17195	AW71_16640	AW74_17290	AW75_16600	AW77_17250
	W	AW68_16860	AW73_16900	AW70_30610	AW67_12230	AW63_16820	AW64_16600	AW47_16535	AW72_15965	AW76_17155	AW71_16600	AW74_17250	AW75_16560	AW77_17210
	Y	AW68_16870	AW73_16910	AW70_30640	AW67_12210	AW63_16830	AW64_16610	AW47_16545	AW72_15975	AW76_17165	AW71_16610	AW74_17260	AW75_16570	AW77_17220
	Z	AW68_16875	AW73_16915	AW70_30650	AW67_12200	AW63_16835	AW64_16615	AW47_16550	AW72_15980	AW76_17170	AW71_16615	AW74_17265	AW75_16575	AW77_17225
sth	*A*	AW68_19705	AW73_19755	AW70_36120	AW67_6690	AW63_19670	AW64_19445	AW47_19405	AW72_18760	AW76_20100	AW71_19680	AW74_20165	AW75_19275	AW77_20055
	B	AW68_19710	AW73_19760	AW70_36130	AW67_6680	AW63_19675	AW64_19450	AW47_19410	AW72_18765	AW76_20105	AW71_19685	AW74_20170	AW75_19280	AW77_20060
	C	AW68_19715	AW73_19765	AW70_36140	AW67_6670	AW63_19680	AW64_19455	AW47_19415	AW72_18770	AW76_20110	AW71_19690	AW74_20175	AW75_19285	AW77_20065
	D	AW68_19720	AW73_19770	AW70_36150	AW67_6660	AW63_19685	AW64_19460	AW47_19420	AW72_18775	AW76_20115	AW71_19695	AW74_20180	AW75_19290	AW77_20070
	E	AW68_19725	AW73_19775	AW70_36160	AW67_6650	AW63_19690	AW64_19465	AW47_19425	AW72_18780	AW76_20120	AW71_19700	AW74_20185	AW75_19295	AW77_20075
std	*A*	AW68_04375	AW73_04375	AW70_8430	AW67_35350	AW63_04640	AW64_04390	AW47_04585	AW72_04355	AW76_04575	AW71_04690	AW74_04750	AW75_04280	AW77_04485
	B	AW68_04380	AW73_04380	AW70_8440	AW67_35340	AW63_04645	AW64_04395	AW47_04590	AW72_04360	AW76_04580	AW71_04695	AW74_04755	AW75_04285	AW77_04490
	C	AW68_04385	AW73_04385	AW70_8450	AW67_35330	AW63_04650	AW64_04400	AW47_04595	AW72_04365	AW76_04585	AW71_04700	AW74_04760	AW75_04290	AW77_04495
	D	AW68_04390	AW73_04390	AW70_8460	AW67_35320	AW63_04655	AW64_04405	AW47_04600	AW72_04370	AW76_04590	AW71_04705	AW74_04765	AW75_04295	AW77_04500
csg	*A*	AW68_14120	AW73_14110	AW70_26050	AW67_17430	AW63_14380	AW64_13860	AW47_14080	AW72_13580	AW76_14700	AW71_13615	AW74_14465	AW75_13995	AW77_14770
	B	AW68_14125	AW73_14115	AW70_26060	AW67_17420	AW63_14385	AW64_13865	AW47_14085	AW72_13585	AW76_14705	AW71_13620	AW74_14470	AW75_14000	AW77_14775
	C	AW68_14115	AW73_14105	AW70_44400	1850980-1850654	AW63_14375	AW64_13855	AW47_14075	AW72_13575	AW76_14695	AW71_13610	AW74_14460	AW75_13990	AW77_14765
	D	AW68_14130	AW73_14120	AW70_26070	AW67_17410	AW63_14390	AW64_13870	AW47_14090	AW72_13590	AW76_14710	AW71_13625	AW74_14475	AW75_14005	AW77_14780
	E	AW68_14135	AW73_14125	AW70_26080	AW67_17400	AW63_14395	AW64_13875	AW47_14095	AW72_13595	AW76_14715	AW71_13630	AW74_14480	AW75_14010	AW77_14785
	F	AW68_14140	AW73_14130	AW70_26090	AW67_17390	AW63_14400	AW64_13880	AW47_14100	AW72_13600	AW76_14720	AW71_13635	AW74_14485	AW75_14015	AW77_14790
	G	AW68_14145	AW73_14135	AW70_26100	AW67_17380	AW63_14405	AW64_13885	AW47_14105	AW72_13605	AW76_14725	AW71_13640	AW74_14490	AW75_14020	AW77_14795
tcf	*A*	AW68_18035	AW73_18080	AW70_32920	AW67_9930	AW63_18000	AW64_17775	AW47_17715	AW72_17165	AW76_18415	AW71_18060	AW74_18500	AW75_17665	AW77_18370
	B	AW68_18030	AW73_18075	AW70_32910	AW67_9940	AW63_17995	AW64_17770	AW47_17710	AW72_17160	AW76_18410	AW71_18055	AW74_18495	AW75_17660	AW77_18365
	C	AW68_18025	AW73_18070	AW70_32900	AW67_9950	AW63_17990	AW64_17765	AW47_17705	AW72_17155	AW76_18405	AW71_18050	AW74_18490	AW75_17655	AW77_18360
	D	AW68_18020	AW73_18065	AW70_32890	AW67_9960	AW63_17985	AW64_17760	AW47_17700	AW72_17150	AW76_18400	AW71_18045	AW74_18485	AW75_17650	AW77_18355
stj	*A*	AW68_19840	AW73_19890	AW70_36370	AW67_6440	AW63_19805	AW64_19580	AW47_19540	-	-	AW71_19800	AW74_20300	-	-
	B	AW68_19835	AW73_19885	AW70_36360	AW67_6450	AW63_19800	AW64_19575	AW47_19535	-	-	AW71_19795	AW74_20295	-	-
	C	AW68_19830	AW73_19880	AW70_36350	AW67_6460	AW63_19795	AW64_19570	AW47_19530	-	-	AW71_19790	AW74_20290	-	-
	STM4574	AW68_19825	AW73_19875	AW70_36340	AW67_6470	AW63_19790	AW64_19565	AW47_19525	-	-	AW71_19785	AW74_20285	-	-
	STM4575	AW68_19820	AW73_19870	AW70_36330	AW67_6480	AW63_19785	AW64_19560	AW47_19520	-	-	AW71_19780	AW74_20280	-	-
mrk	*A*	-	-	-	-	-	-	-	-	-	-	-	AW75_11755	AW77_12415
	B	-	-	-	-	-	-	-	-	-	-	-	AW75_11760	AW77_12420
	C	-	-	-	-	-	-	-	-	-	-	-	AW75_11765	AW77_12425
	F	-	-	-	-	-	-	-	-	-	-	-	AW75_11775	AW77_12435

Supplemental Figure and Tables

Fig. S1. The SPI-7-like ICESe3 in Montevideo clades II and III showing high conservation. Integrase/shufflon – light blue, DNA helicase – blue, DNA polymerase *umuC* – dark purple, toxin/antitoxin *cbtA* – pink, conjugal transfer *tra* – light green/dark green, adhesins – red, type IVB pili – purple, DNA topoisomerase – yellow, plasmid partitioning *parB* – salmon.

Table S1. List of strains sequenced in this study, NCBI accession numbers, and genome size for complete, closed genomes (indicated by CP number in bold).

Table S2. Mobile genetic elements of Montevideo. Prophages are identified by PHASTER and visual inspection along with nucleotide locations listed. PHASTER hits are shown with closest 3 prophages with shared genes in parentheses. Phage regions are listed as SM Φ1-14 in order of appearance on the Montevideo pangenome.

Table S3. Metabolic island differences among the Montevideo clades as well as locus tag IDs for genes delineating each metabolic island.

Table S4. Locus tag IDs for all secreted effectors in the Montevideo strains sequenced identified by BLAST analysis using the virulence finder database (VFDB), as well as function if known.

Table S5. Locus tag IDs of Montevideo virulence genes identified by BLAST analysis using the virulence finder database (VFDB), as well as function if known.

Table S6. Locus tag IDs (if annotated, otherwise genome coordinates are indicated) of genes within *fim* operons identified in Montevideo sequences.
